# Downregulation of the inflammatory network in senescent fibroblasts and aging tissues of the long‐lived and cancer‐resistant subterranean wild rodent, *Spalax*

**DOI:** 10.1111/acel.13045

**Published:** 2019-10-11

**Authors:** Amani Odeh, Maria Dronina, Vered Domankevich, Imad Shams, Irena Manov

**Affiliations:** ^1^ Department of Evolutionary and Environmental Biology Faculty of Natural Sciences University of Haifa Haifa Israel; ^2^ Institute of Evolution University of Haifa Haifa Israel; ^3^ Present address: Tel Aviv University Tel Aviv Israel

**Keywords:** cellular senescence, DNA damage, DNA repair, interleukin‐1 alpha (IL1α), nuclear factor κB (NF‐κB), senescence‐associated secretory phenotype (SASP), *Spalax*

## Abstract

The blind mole rat (*Spalax*) is a wild, long‐lived rodent that has evolved mechanisms to tolerate hypoxia and resist cancer. Previously, we demonstrated high DNA repair capacity and low DNA damage in *Spalax* fibroblasts following genotoxic stress compared with rats. Since the acquisition of senescence‐associated secretory phenotype (SASP) is a consequence of persistent DNA damage, we investigated whether cellular senescence in *Spalax* is accompanied by an inflammatory response. *Spalax* fibroblasts undergo replicative senescence (RS) and etoposide‐induced senescence (EIS), evidenced by an increased activity of senescence‐associated beta‐galactosidase (SA‐β‐Gal), growth arrest, and overexpression of p21, p16, and p53 mRNAs. Yet, unlike mouse and human fibroblasts, RS and EIS *Spalax* cells showed undetectable or decreased expression of the well‐known SASP factors: interleukin‐6 (IL6), IL8, IL1α, growth‐related oncogene alpha (GROα), SerpinB2, and intercellular adhesion molecule (ICAM‐1). Apparently, due to the efficient DNA repair in *Spalax*, senescent cells did not accumulate the DNA damage necessary for SASP activation. Conversely, *Spalax* can maintain DNA integrity during replicative or moderate genotoxic stress and limit pro‐inflammatory secretion. However, exposure to the conditioned medium of breast cancer cells MDA‐MB‐231 resulted in an increase in DNA damage, activation of the nuclear factor κB (NF‐κB) through nuclear translocation, and expression of inflammatory mediators in RS *Spalax* cells. Evaluation of SASP in aging *Spalax* brain and intestine confirmed downregulation of inflammatory‐related genes. These findings suggest a natural mechanism for alleviating the inflammatory response during cellular senescence and aging in *Spalax*, which can prevent age‐related chronic inflammation supporting healthy aging and longevity.

## Graphical abstract

The uncoupling of senescence phenotype and the inflammatory component of SASP was discovered in long‐lived blind mole rat, *Spalax*, adapted to extreme environments. Cellular senescence in *Spalax*, evidenced by increased SA‐β‐Gal activity, upregulation of p21/p16/p53, is not accompanied by accumulation of DNA damage nor by secretion of SASP factors. We suggest that genome integrity, inhibition of NF‐κB nuclear translocation, and IL1α suppression are involved in overcoming the inflammatory response in aging Spalax.

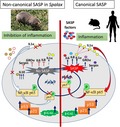

## 1 INTRODUCTION

Ten years have passed since senescence‐associated secretory phenotype (SASP), a complex of predominantly pro‐inflammatory factors secreted by aging cells, was first described (Coppe et al., 2008; Kuilman & Peeper, 2009). The seminal works on this topic heralded a new era of understanding the involvement of cellular senescence in age‐related pathology. Senescent cells lose their ability to divide, inhibiting the propagation of mutations to the next cell generation in response to telomere shortening, DNA damage, or oncogenic stimulus. Notwithstanding the physiological role of senescence in preventing malignant transformation (Campisi, 2013), senescent cells accumulate in damaged or aging tissues and influence the surrounding cells through SASP factors (Lasry & Ben‐Neriah, 2015). SASP contributes to tissue repair and reinforcement of senescence in damaged cells (Acosta et al., 2013); however, it is involved in the maintenance of the pro‐inflammatory microenvironment, resulting in the acquisition of sterile inflammation, leading to age‐associated diseases including cancer (Franceschi & Campisi, 2014; Tchkonia, Zhu, van Deursen, Campisi, & Kirkland, 2013).

The DNA damage response (DDR), the main inducer of SASP, is activated in response to DNA lesions that trigger DNA repair programs (Rodier et al., 2009). If DNA damage is not resolved, cells undergo a nonreversible proliferative arrest associated with a persistently active DDR, causing the secretion of inflammatory factors. Data indicate that detrimental effect of senescent cells on surrounding tissues and the whole body is attributed to both the effects of arrested cells themselves (McGlynn et al., 2009) and secretion of SASP factors (Tchkonia et al., 2013). Therefore, inhibition of SASP is now considered an alternative to senolytic therapy to target the deleterious effects of senescent cells (Georgilis et al., 2018; Tchkonia et al., 2013).

Recent evidence has indicated that SASP and cell cycle arrest are regulated by different signaling molecules. It was demonstrated that senescence may occur without genotoxic stress, for example, by overexpression of p16^Ink4a^, the regulator of retinoblastoma protein (Coppe, Desprez, Krtolica, & Campisi, 2010). The secretion of interleukin‐6 (IL6), a key SASP factor, was shown to be unrelated to senescence arrest, but rather caused by persistent DDR activation in a p53‐independent manner (Rodier et al., 2009). Rapamycin, the inhibitor of mammalian target of rapamycin, suppresses SASP but not cell cycle arrest (Laberge et al., 2015; Wang et al., 2017).

Developmental senescence is characterized by common markers of senescent cells, such as senescence‐associated β‐galactosidase (SA‐β‐Gal), p21, and p15; however, it is not accompanied by DDR and secretion of major SASP factors (Munoz‐Espin et al., 2013; Storer et al., 2013). Thus, in some physiological contexts, senescence develops without an inflammatory response, and SASP in its conventional form (“canonical SASP”) is not mandatory for acquisition of senescence.

The blind mole rat, *Spalax*, is a long‐lived, cancer‐resistant subterranean mammal that has evolved efficient survival mechanisms and adaptations to stressful conditions in its underground hypoxic and hypercapnic environment (Avivi et al., 2010; Schulke et al., 2012; Shams, Avivi, & Nevo, 2005). *Spalax* evolved unique capability to maintain oxygen homeostasis by rapid erythrocyte production, which is achieved by an adaptive elevation in Epo mRNA expression and the ability to extract iron from ferritin (Iancu, Arad, Shams, & Manov, 2014; Shams, Nevo, & Avivi, 2005). These and other features that *Spalax* evolved during 40 million years of evolution underground allow it to cope with continuous stress and maintain strong cellular and tissue homeostasis, apparently providing resistance to cancer and a prolonged lifespan. Recently, we demonstrated that *Spalax* skin fibroblasts successfully resist genotoxic stress through effective DNA repair, compared with fibroblasts of *Rattus* (Domankevich, Eddini, Odeh, & Shams, 2018). These data explain, at least in part, *Spalax* resistance to chemical‐induced carcinogenesis observed earlier (Manov et al., 2013). In this study, we focused on cellular senescence in *Spalax* fibroblasts. Two models of cellular senescence, replicative senescence (RS) and etoposide‐induced senescence (EIS), were used to evaluate proliferative arrest and senescent secretory phenotype in *Spalax* primary fibroblasts, in comparison with mouse and human cells. Our data revealed neither substantial DNA damage nor enhanced expression of well‐characterized pro‐inflammatory SASP factors: interleukin‐6 (IL6), IL8, IL1α, growth‐related oncogene alpha (GROα), SerpinB2, intercellular adhesion molecule (ICAM‐1), and cyclooxygenase‐2 (Cox2). Reduced mRNA expression of pro‐inflammatory SASP representatives was also found in aging *Spalax* tissues. The secretory phenotype of *Spalax* senescent cells that lack basic inflammatory factors, termed here “noncanonical SASP,” was investigated, and several molecular aspects of its regulation in *Spalax* were evaluated.

## 2 RESULTS

### 2.1 *Spalax* fibroblasts, similarly to human and mouse fibroblasts, undergo replicative and etoposide‐induced senescence

Fibroblasts were subjected to serial passages, and senescence phenotype was defined as a state in which most of the cells have acquired an enlarged, flattened morphology, the number of dividing cells significantly decreased, and most cells exhibit positive SA‐β‐Gal. Similar to mouse cells, *Spalax* fibroblasts became senescent at passages 5–8, while for human fibroblasts, more than 40 passages were required to bring about senescence (the cells completely lost their ability to divide at passage #55–60). Noteworthy, replicative capacity of mammalian fibroblasts in culture does not reflect species longevity but correlates well with body size (Lorenzini, Tresini, Austad, & Cristofalo, 2005). Therefore, human fibroblasts took more divisions to achieve senescence. Late‐passage cells (#6–8 for *Spalax*; #5‐6 for mice; and above # 46 for human) showed a significant increase in SA‐β‐Gal activity compared with early‐passage ones (Figure S1A,B). The population doubling rates of young fibroblasts of *Spalax*, mice, and human were the same; cells underwent 3.125 divisions, and the number of cells increased from 2.5 × 10^4^ to 250 × 10^4^ in 96 hr. Thus, the *in vitro* experimental conditions that we used provided the same proliferative activity for growing human, *Spalax,* and mouse fibroblasts (Figure S1C). Proliferative capabilities gradually decreased with aging. Once the fibroblasts reached the stage of replicative aging, they ceased to divide, remained alive, and could be subjected to further passages in a 1:1 ratio.

**Figure 1:**
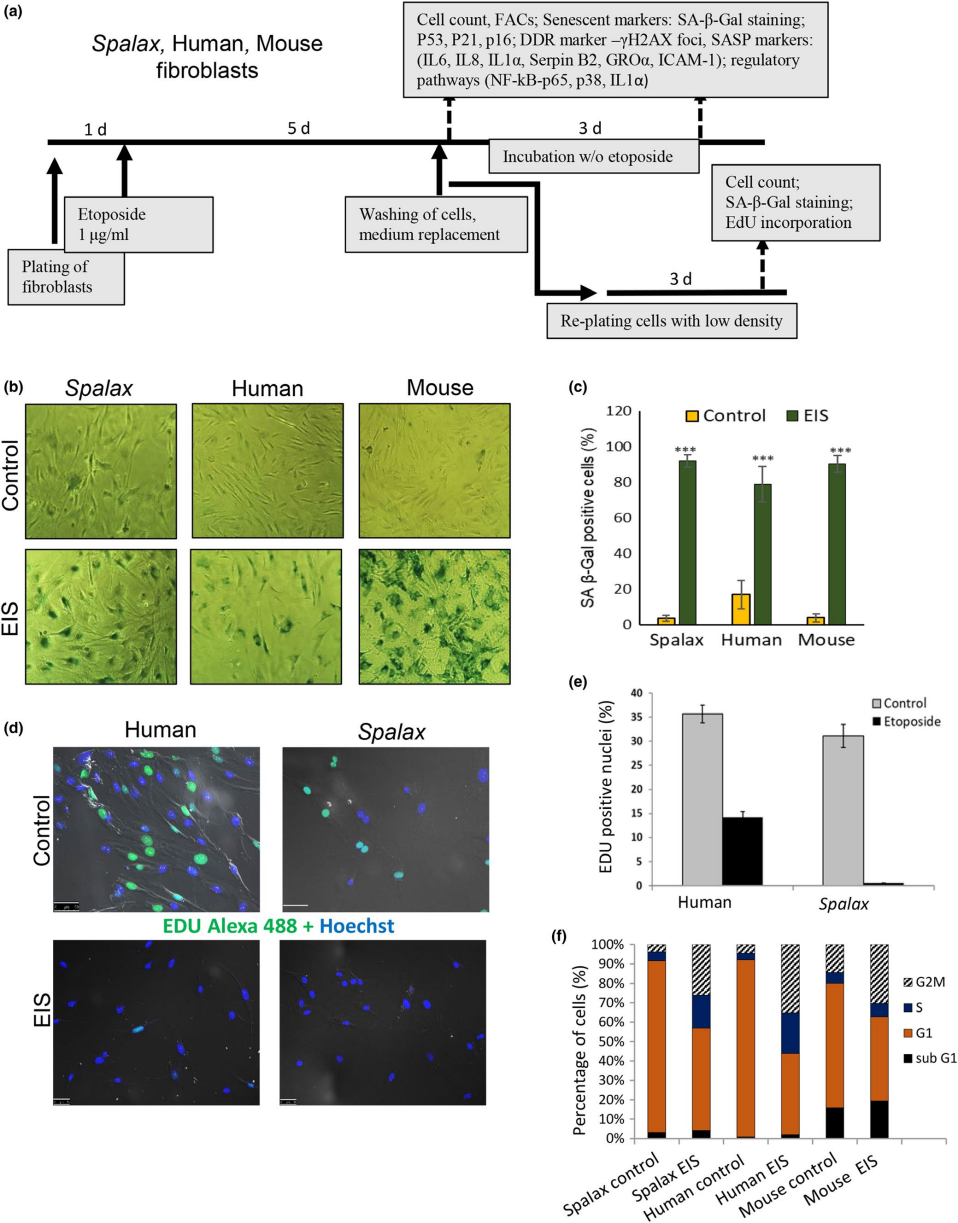
Etoposide‐induced senescence (EIS). (a) Schematic illustration of the experimental design. (b) SA‐β‐Gal staining representative images. Fibroblasts were treated with DMSO (control) or with 1 μg/ml etoposide for 5 days, washed, and incubated for an additional 3 days without etoposide. (c) Percentage of SA‐β‐Gal‐positive cells calculated from at least 300 cells in four independent fields for each biological repeat (*n* = 3) in triplicate (*Spalax* and mouse cells were isolated from three independent individuals). The mean value of each species is compared to its control cells (DMSO‐treated), *** *p* < .001. (d) Representative fluorescence images demonstrating the levels of EdU incorporation in *Spalax* and human untreated and etoposide‐treated cells. (e) Quantitative data showing the % of EdU‐positive nuclei in untreated and etoposide‐treated *Spalax* and human fibroblasts. Experiments were repeated two times (*n* = 2) in triplicate. (f) Flow cytometry analysis showing the percentage of cells in cell cycle stages. Values represent the averages of at least two independent experiments in triplicate. EdU, 5‐ethynyl‐2‐deoxyuridine

To investigate DNA damage‐induced senescence, fibroblasts were subjected to etoposide, a chemotherapeutic compound that induces DNA damage through the inhibition of DNA topoisomerase II (Yang et al., 2009). Etoposide induces cellular senescence or apoptosis, depending on the concentration used. *Spalax* and mouse fibroblasts (passage #2–4) and human primary fibroblasts (passage #3–6) were treated with either 1 µg/ml etoposide (Leontieva & Blagosklonny, 2010) or DMSO (control) for 5 days. The cells were then washed and either collected for analysis or replated and incubated for an additional 3 days, followed by SA‐β‐Gal activity staining and EdU (5‐ethynyl‐2‐deoxyuridine) incorporation assay (a detailed scheme of the experiments is presented in Figure 1a). The number of SA‐β‐Gal‐positive cells was significantly increased in EIS fibroblasts from *Spalax*, mice, and human, compared with the control (Figure 1b,c). *Spalax* fibroblasts exposed to etoposide exhibited an irreversible growth arrest, confirmed by decreased EdU incorporation following low‐density replating and incubation for an additional 3 days (in the absence of etoposide; Figure 1d). The percentages of EdU‐positive nuclei (representing proliferating cells) were 35.65 ± 5.17% and 31.09 ± 8.18% among untreated human and *Spalax* cells, respectively (Figure 1e). Among etoposide‐treated human cells, only 14.21 ± 10.5% EdU‐positive nuclei were observed (*p* ≤ .01 *vs*. control), whereas no EdU‐positive nuclei were detected among EIS *Spalax* fibroblasts. Flow cytometry has shown that etoposide‐induced proliferative arrest was due to an accumulation of cells in the S‐G2/M phases of the cell cycle (Figure 1f).

### 2.2 The p53–p21–p16 tumor suppressor pathways are involved in the establishment of senescence in *Spalax* fibroblasts

**Figure 2:**
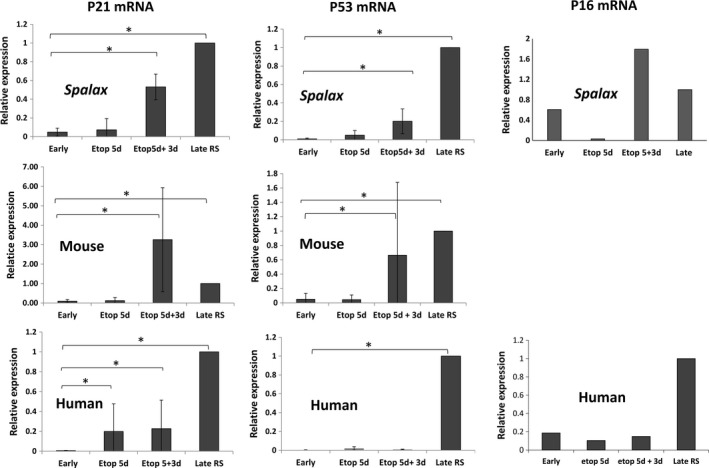
Expression of p21, p16, and p53 markers in senescent fibroblasts. The levels of mRNA expression were quantified in young cells (early‐passage fibroblasts) and senescent cells (late‐passage or etoposide‐treated fibroblasts) by using relative qRT–PCR; data of p21 and p53 mRNA levels are presented as mean ± *SD* of three independent experiments (*n* = 3, cells from three different individuals of *Spalax* and mice). * *p* < .05 depicts significant differences from early passage. p16 expression (representative data of two independent experiments are shown). RS, replicative senescence; Etop, etoposide

The p53, p21, and p16 proteins are key factors in cellular senescence. Thus, we tested whether mRNA expression of these markers is increased in *Spalax* fibroblasts undergoing RS and EIS. As shown in Figure 2, the mRNA levels of p53, which were undetectable in young *Spalax* cells, were significantly upregulated in late‐passage fibroblasts. Similarly, the levels of p21 and p16 mRNA increased in *Spalax* RS cells. Etoposide treatment resulted in a marked increase in p21 and p16 mRNA levels in *Spalax* fibroblasts, 3 days following etoposide withdrawal (5d + 3d). Likewise, late‐passage human and mouse fibroblasts showed an increase in p53/p21 mRNA levels and p16 (human); changes in p53 and p16 mRNA levels were less prominent in human EIS cells compared with *Spalax*.

### 2.3 No increase in γH2AX foci number in RS and EIS fibroblasts of *Spalax*

**Figure 3:**
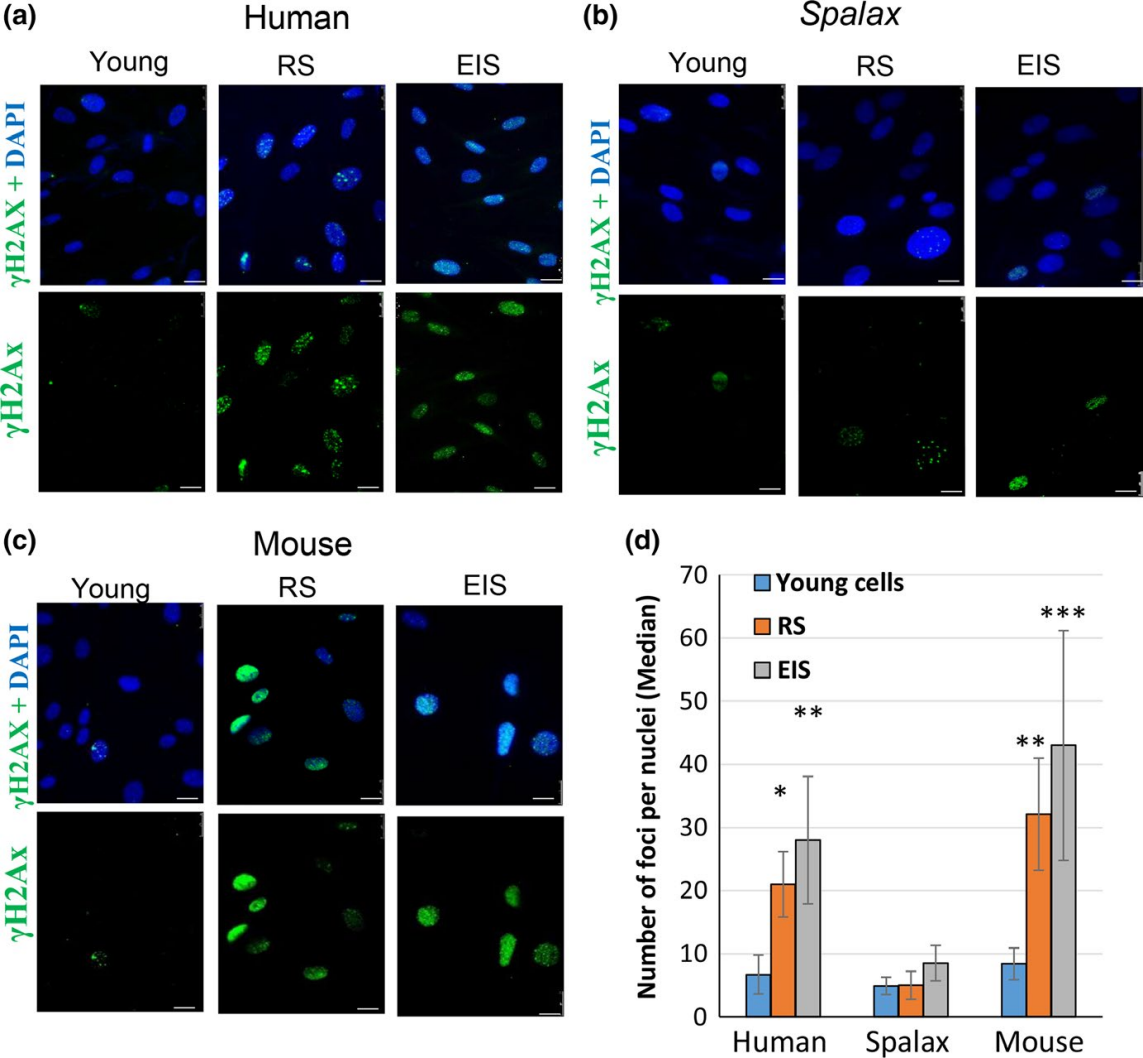
Distinct DDR in *Spalax*, human, and mouse fibroblasts undergoing replicative senescence (RS) and etoposide‐induced senescence (EIS). To induce senescence, young cells were treated with 1 μg/ml of etoposide for 5 days; then, etoposide was washed out and cells were replated and incubated for an additional 48 hrs. Thereafter, cells were fixed and stained with anti‐phospho‐H2AX antibody and counterstained with DAPI. Cells' nuclei were visualized under fluorescent microscope. The images were used for quantification using FociCounter software (a minimum of 250 nuclei per sample were analyzed). Representative images of γH2AX (Ser139) foci in the nuclei of young, RS, and EIS human (a), *Spalax* (b), and mouse (c) fibroblasts are demonstrated. (d) A quantitative analysis of foci numbers. Data are presented as median of 250 nuclei counted for each species, assessed in three independent experiments (*n* = 3). *** *p* ≤ .001, ** *p* ≤ .01, * *p* ≤ .05, differences between the groups of young and senescent nuclei. Scale bars are 25 µm

Persistent DNA damage is required to induce an inflammatory response and SASP in senescent cells (Rodier et al., 2009; Wang et al., 2009). *Spalax* fibroblasts demonstrated resistance to acute DNA damage and high DNA repair capability (Domankevich et al., 2018); therefore, we investigated the level of DDR in *Spalax* fibroblasts undergoing replicative and premature senescence. Since phosphorylated H2AX histone (γH2AX) is a reliable indicator of DNA double‐strand breaks (DSBs) (Sharma, Singh, & Almasan, 2012), we used γH2AX foci quantification in our experiments. A significant increase in γH2AX foci number was observed in human and mouse RS and EIS fibroblasts compared with young cells (Figure 3a,c,d); however, in *Spalax* RS and EIS fibroblasts, we did not find notable changes in the number of foci *vs*. young cells (Figure 3b,d). Notably, the lack of DSB detection in *Spalax* cells is unlikely to be associated with low specificity of anti‐human γH2AX: the sequence identity between *Spalax* and human H2AX is 99%, and that for the immunogenic epitope is 100%.

### 2.4 The reduced DNA damage in *Spalax* fibroblasts exposed to etoposide is likely due to the high efficiency of DNA repair

**Figure 4:**
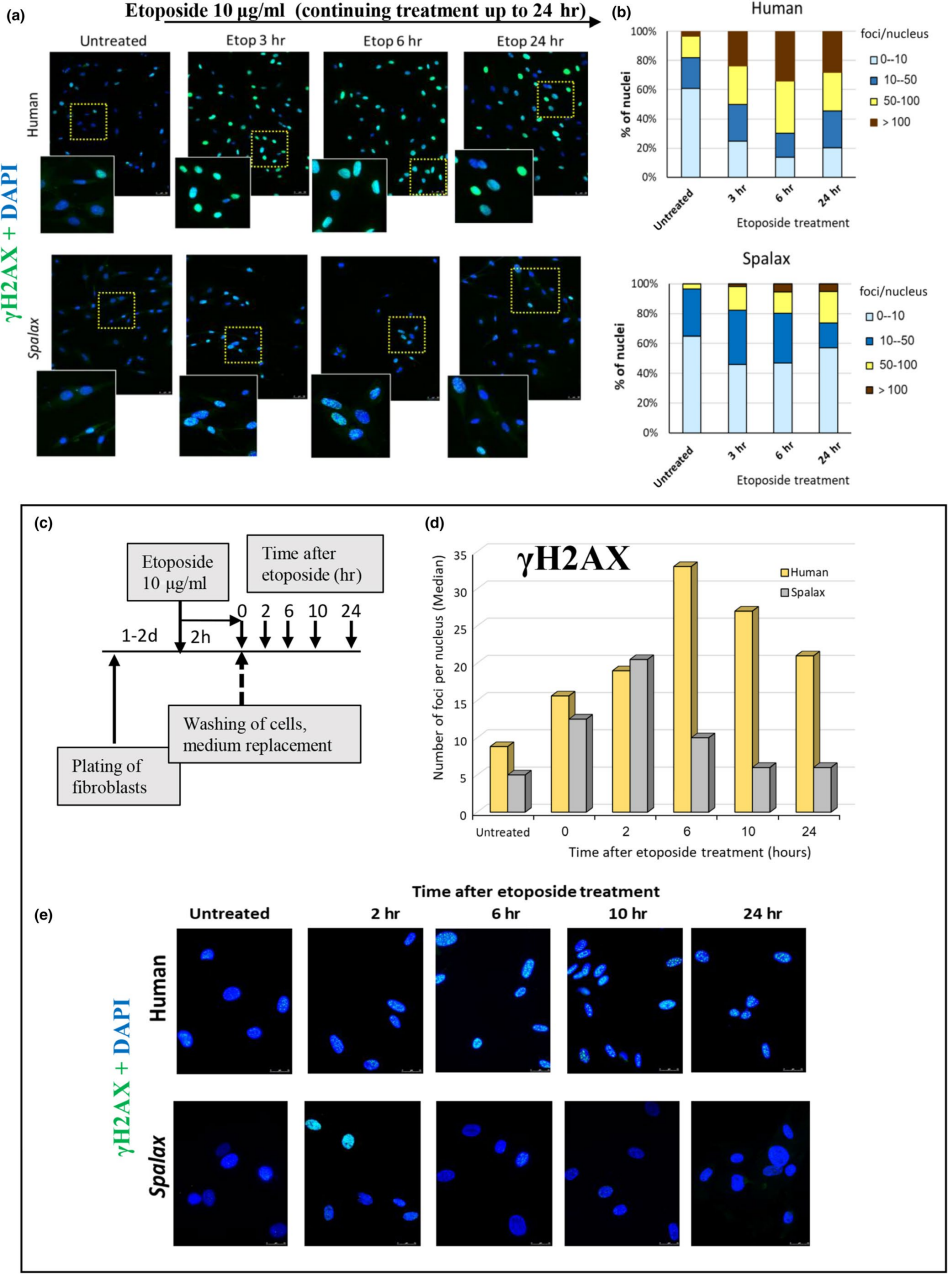
Dynamics of DNA damage/ repair in the responses to continuous etoposide exposure to 24‐hr (a + b) and short 2‐hr treatment, followed by recovery (c + d + e): *Spalax* *vs*. human. (a) Fibroblasts were exposed to 10 μg/ml etoposide. After 3, 6, and 24 hr, cells were fixed and stained with anti‐phospho‐H2AX to evaluate the rates of DSBs. Representative images are presented. A magnified view of the boxed regions is shown, highlighting the differences in the number of γH2AX foci in human and *Spalax*. Scale bars are 50 µm. (b) Distribution of foci (% of total nuclei) in fibroblasts of human and *Spalax* untreated and treated with etoposide for 3, 6, and 24 hr. A total of 250 or more nuclei were evaluated in each sample. (c) Schematic representation of experiment. (d) The dynamic of DNA damage/repair in human and *Spalax* cells after treatment with etoposide is estimated as the percentage of nuclei with different numbers of DSB foci (at least 250 nuclei per sample were analyzed). (e) Representative images showing differences in DNA damage/repair dynamics in human and *Spalax* fibroblasts after treatment with etoposide. Experiments were repeated two times (*n* = 2) in triplicate. Etop, etoposide

To evaluate whether low DNA damage in *Spalax* senescent cells is associated with effective DNA repair, we used two approaches: (A) *Spalax* and human fibroblasts were treated with etoposide 10µg/ml for 3, 6, and 24 hr to compare the dynamics of DNA damage (Figure 4a,b). Untreated cells of both species revealed the same number of nuclei with less than 10 foci/nucleus. The high appearance of cells with intensive DNA damage (50–100 foci) and massive pan‐nuclear DSBs (scored as more than 100 foci) was observed in human cells already 3 hr after the onset of exposure and remained at about this level for up to 24 hr. In *Spalax* cells, the number of γH2AX foci increased compared with untreated cells, but most of the damaged nuclei contained 10–50 foci, while the nuclei with low DSBs (<10 foci) were 40%–50% (compared to 10%–20% in human cells). These results showed that *Spalax* is highly resistant to etoposide genotoxicity compared to human cells. (B) To assess and to compare the ability to repair DNA, *Spalax* and human fibroblasts were treated with 10 μg/ml etoposide for 2 hr; then, the medium was replaced, and the cells were incubated for specified periods of time without etoposide, to allow DSB repair (Figure 4c,d,e). The number of γH2AX foci in the nuclei of human fibroblasts gradually increased and reached maxima at 6 hr after etoposide withdrawing. Then, the DSB level decreased, but did not drop to the level observed before treatment, even after 24 hr (Figure 4d). In *Spalax* cells, the highest DSB value was observed 2 hr after etoposide treatment (at levels similar to human fibroblast levels); then, the DSB level decreased, and after 10 hr, it reached the level of untreated cells. These data showed that *Spalax* cells can maintain DNA integrity under replicative or moderate genotoxic stress, which may explain the failure of SASP in senescence.

### 2.5 The lack of mRNA transcription of major inflammatory mediators is a hallmark of the noncanonical SASP in *Spalax* senescent fibroblasts

**Figure 5:**
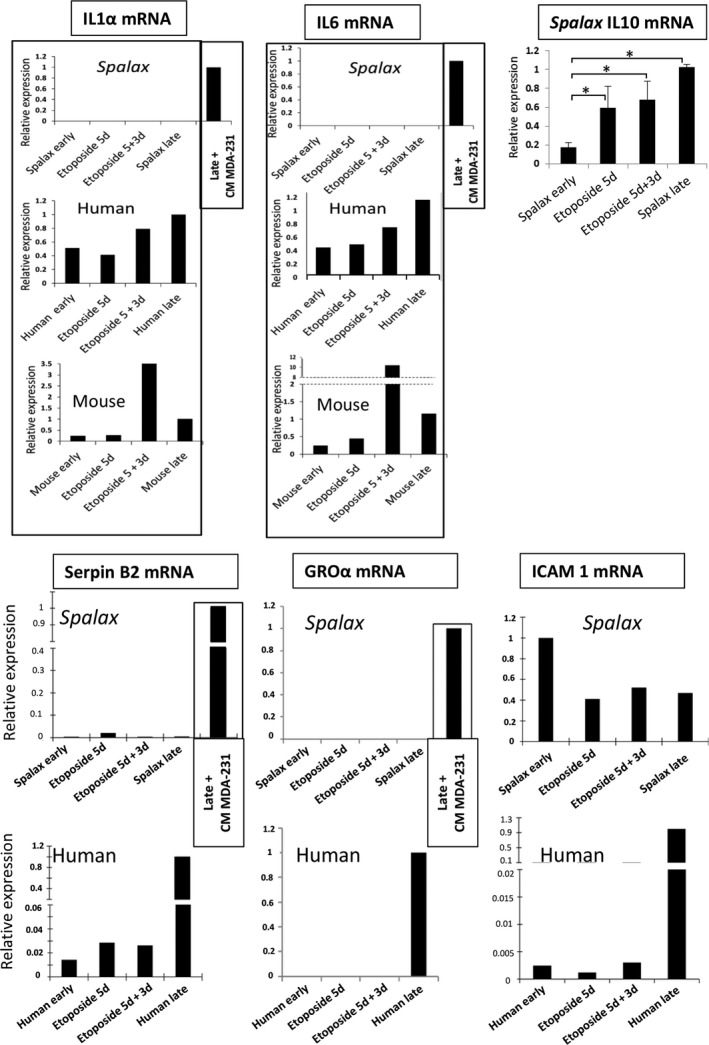
Levels of mRNA expression of the main inflammatory mediators in young, RS, and EIS fibroblasts of *Spalax*, human, and mice. The mRNA expression rates were quantified by using qRT–PCR. Representative data for IL1α, IL6, SerpinB2, GROα, and ICAM‐1 are shown. Additional independent biological repeats [cells isolated from different animals (*Spalax* and mice) or different experiments (human cells)] are presented in Figures S2a, S3, and S4. Treatment of *Spalax* fibroblasts with MDA‐MB‐231 CM was used for the induction of pro‐inflammatory cytokines as a positive control. The top right panel demonstrates the increase in IL10 mRNA in *Spalax* senescent cells. Bars represent mean ± *SD* of three independent biological experiments (*n* = 3; * *p* ≤ .05). Note: The IL10 mRNA expression was undetectable in human and mouse cells. Etop, etoposide; RS, replicative senescence

Senescence‐associated secretory phenotype is a distinguishing feature of senescent cells, and it usually accompanies proliferative arrest. The correlation between DDR and SASP is well defined (Kang et al., 2015; Rodier et al., 2009). While DDR is activated in a short time (minutes) after DNA damage, and p53 transcription stabilization takes several hours, several days are required to achieve maximum secretion of SASP factors, such as IL6 and IL8 (Coppe et al., 2008; Rodier et al., 2009). Our results show that *Spalax* senescent fibroblasts undergoing either RS or premature EIS did not show an increase in the number of γH2AX foci, most likely due to the higher capacity of *Spalax* to repair DNA damage. Since the activation of DDR is required for the induction and maintenance of SASP, we assessed the levels of mRNA expression of several known inflammatory mediators. As demonstrated, the mRNA expression of IL6 and IL1α, the key factors of SASP, increased in human and mouse RS and EIS cells (Figure 5; Figures S2 and S3). The increased levels of IL6 production by human RS fibroblasts *vs*. young cells was confirmed by using an ELISA kit (Figure S2B). In contrast, mRNA expressions of IL6 and IL1α in *Spalax* were undetectable (Figure 5; Figure S4). The mRNA expression of the pro‐inflammatory factors GROα and SerpinB2, like IL6 and IL1α, was not observed in *Spalax* RS cells (Figure 5; Figure S4), while it has been robustly increased in human RS fibroblasts (Figure 5). In addition, the expression of ICAM‐1 mRNA, an important marker of inflammation that was activated in human RS cells, was markedly reduced in senescent *Spalax* cells compared to young cells (Figure 5; Figure S4). Consistently, the expression of IL8 and Cox2 mRNAs was undetectable in *Spalax* senescent fibroblasts (Figure S4). Interestingly, both RS and EIS in *Spalax* fibroblasts were associated with an increase in IL10 mRNA (Figure 5), an anti‐inflammatory cytokine (Murray, 2005); however, it was undetectable in all human or mouse cells (data not shown).

### 2.6 Soluble factors of MDA‐MB‐231 conditioned medium induce strong inflammatory response in *Spalax* RS fibroblasts

Accumulating data suggest that SASP or inflammatory cytokines may trigger DDR and inflammatory signaling in the surrounding microenvironment, an effect known as “bystander” senescence (Hubackova, Krejcikova, Bartek, & Hodny, 2012; Sapega et al., 2018). Multiple inflammatory factors secreted by aggressive metastatic MDA‐MB‐231 breast cancer cells, including extremely high concentrations of IL1β, were able to induce NF‐κB signaling and secretion of inflammatory cytokines and chemokines in bystander cells (Escobar et al., 2015). To determine whether expression of inflammatory factors in *Spalax* RS fibroblasts can be activated by exogenous inflammatory stimuli, we treated *Spalax* late‐passage cells with conditioned medium (CM) of MDA‐MB‐231 cells. Exposure of *Spalax* senescent cells to MDA‐MB‐231 CM resulted in a strong activation of IL6, IL8, IL1α, GROα, SerpinB2, and Cox2 mRNA expression (Figure 5; Figure S4) and a significant increase in γH2AX foci in the nuclei (Figure S5). Thus, *Spalax* has developed effective mechanisms for limiting the inflammatory response during physiological and stress‐induced senescence that are not accompanied by severe DNA damage; however, SASP signaling can be triggered in *Spalax* cells via strong inflammatory stimulation.

### 2.7 Phosphorylation of p65 in RS *Spalax* fibroblasts is not sufficient for its translocation into the nucleus and activation of inflammatory response

**Figure 6:**
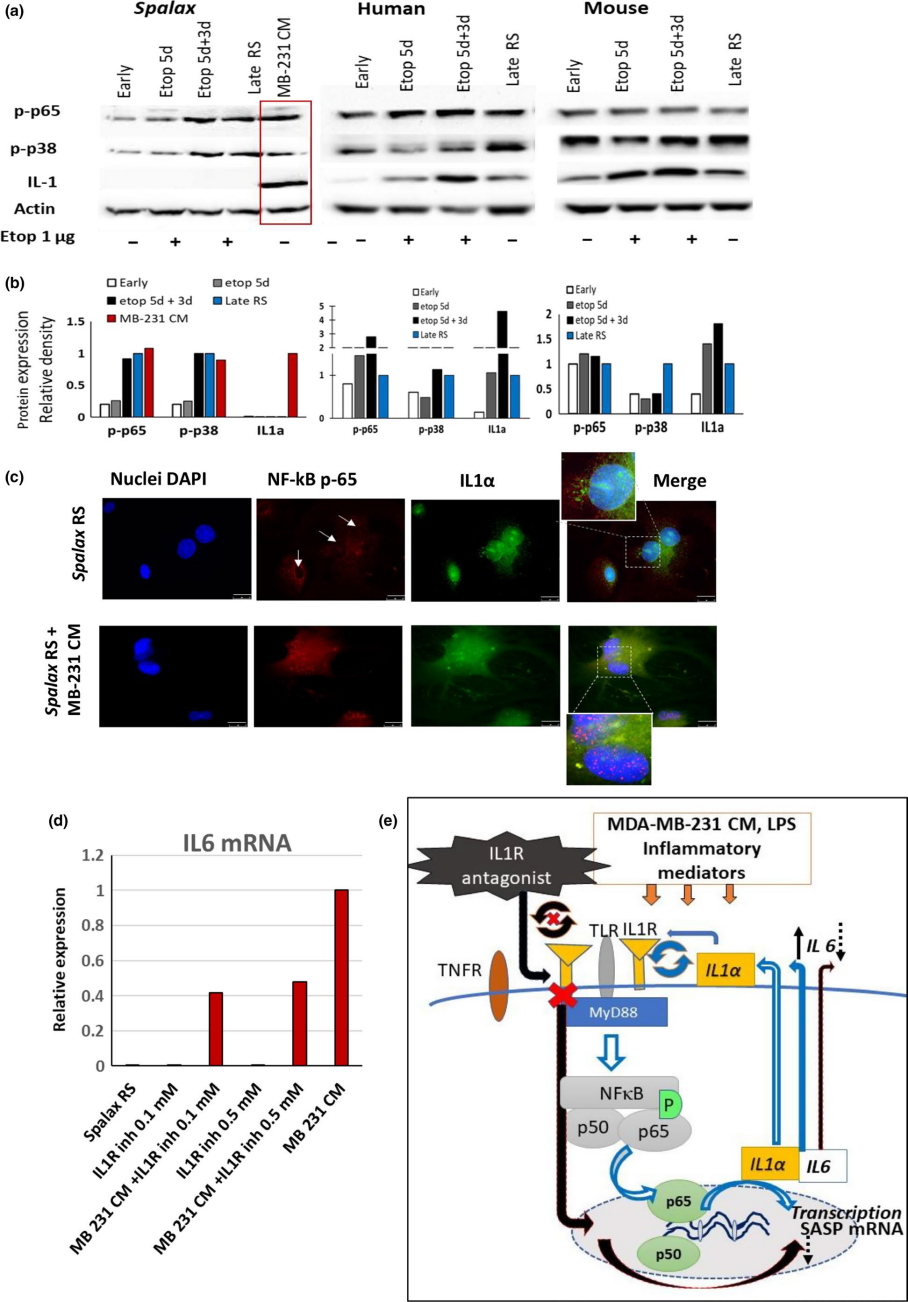
Inhibition of NF‐κB nuclear translocation and suppression of surface‐membrane localization of IL1α are involved in downregulation of the inflammatory response in senescent *Spalax* cells. (a) Representative Western blot analysis demonstrating the protein expression of p‐p65(Ser^536^), p‐p38, and IL1α in *Spalax*, human, and mouse fibroblasts undergoing replicative or etoposide‐induced senescence. Additional independent biological repeats (cells from different genotypes) are shown in Figures S6 and S7. Densitometry quantification of Western blots is presented in (b). (c) Microphotographs showing the localization of NF‐κB‐p65 within the cells and intracellular distribution of IL1α in *Spalax* RS fibroblasts untreated or treated with MDA‐MB‐231 CM. Arrows indicate the absence of NF‐κB‐p65 in the nuclei of RS *Spalax* cells. Inserts present a high magnification of nuclear zone. Scale bars are 25µm. Experiments were repeated two times (*n* = 2). Complimentary microphotographs are presented in Figure S9a. (d) Inhibition of IL1R pathway suppressed the IL6 cytokine mRNA expression induced by MDA‐MB‐231 CM in RS *Spalax* fibroblasts. Cells were treated with MDA‐MB‐231 CM alone or together with the chemical antagonist of IL1R for 24 hr (at the concentrations of 0.1 and 0.5 mM) or with antagonist alone. The IL6 mRNA levels were analyzed by qPCR. (e) A simplified diagram explaining the inhibitory effect of an IL1R antagonist on SASP (IL6) expression in Spalax fibroblasts caused by inflammatory factors of MDA‐MB‐231 CM: The pro‐inflammatory factors of MDA‐MB‐231 CM bind to the corresponding receptors (TLR, IL1R, TNFR) and induce signal transduction pathways leading to the activation of NF‐κB. Toll‐like receptors (TLRs), including IL1R, recruit the receptor‐related protein MyD88, which in turn phosphorylates and activates specific kinases, which leads to the phosphorylation of p65 and its translocation to the nucleus. In response to these events, the expression of SASP factors begins. IL1α is a minor component of SASP, but when it reaches the surface membrane, it binds the IL1R receptor and acts as an SASP enhancer. A chemical antagonist binds the region of IL1R/MyD88 interaction, inhibiting the positive feedback loop of IL1α/NF‐κB, which, in turn, leads to a decrease in the level of IL6 expression (IL6 was used as indicator of SASP)

Despite substantial amounts of data published to date on the molecular pathways responsible for activating SASP during aging, little is known about the mechanisms that can suppress the inflammatory response in senescent cells. We tested the possibility that suppression of SASP in senescent *Spalax* cells is associated with reduced phosphorylation of two important regulatory factors, NF‐κB and p38, both of which are involved in SASP initiation and maintenance (Chien et al., 2011; Orjalo, Bhaumik, Gengler, Scott, & Campisi, 2009). Contrary to our expectations, *Spalax* senescent cells demonstrated increased phosphorylation of p65 NF‐κB (Ser^536^) and p38, similar to mouse and human senescent fibroblasts (Figure 6a,b; Figures S6 and S7). Whereas p65 activation via phosphorylation was observed, the nuclei of *Spalax* RS cells remained mostly devoid of p65 (Figure 6c; Figure S9A). Nevertheless, the treatment of *Spalax* senescent cells with MDA‐MB‐231 CM or lipopolysaccharide (LPS) triggered a strong nuclear localization of p65 (Figure 6c; Figure S9A,B), which is a hallmark of NF‐κB activation (Chien et al., 2011). These data suggest that the absence of inflammatory SASP factors in *Spalax* can be attributed, at least in part, to the inhibition of p65 nuclear translocation.

### 2.8 Downregulation of the inflammatory network in *Spalax* senescent fibroblasts is partially associated with suppression of IL1α cytokine response

IL1α is an inflammatory cytokine and a minor component of SASP, but it is the major player in the regulation of the inflammatory response (Apte et al., 2006; Laberge et al., 2015). The IL1α pathway is upregulated in replicative and induced senescence (Purcell, Kruger, & Tainsky, 2014). The expression and secretion of IL6 and IL8 by human senescent fibroblasts was found to depend on surface membrane‐bound IL1α (Orjalo et al., 2009). We verified the increase in IL1α expression at mRNA and protein levels in human and mouse RS and EIS fibroblasts (Figures 5 and 6; Figures S2, S3, S6, and S7). Neither IL1α mRNA nor protein expression was detected in *Spalax* senescent cells (Figures 5 and 6a,b; Figures S4 and S6). Nevertheless, when stimulated by MDA‐MB‐231 CM, *Spalax* RS fibroblasts demonstrated prominent expression of IL1α mRNA and protein (Figures 5 and 6a,b; Figures S4 and S6). Since it was demonstrated that surface‐bound IL1α is involved in the activation of the inflammatory response, we analyzed intracellular IL1α distribution by immunofluorescence (Figure S8A,B). In untreated RS fibroblasts of *Spalax*, IL1α was poorly represented and concentrated mainly around the nuclei. No surface membrane‐bound IL1α was found (Figure [Link], [Link], [Link]A, upper panels). However, with MDA‐MB‐231 CM, IL1α was distributed through the cells, reaching the surface membrane (Figure S8A, low panels), and propagated evenly among different cellular compartments (Figure S8A,B). Similar results were obtained when *Spalax* RS cells were stimulated with LPS (Figure S9B). To further confirm that surface‐bound IL1α, stimulated by inflammatory stimuli in *Spalax* RS cells, is involved in suppressing the secretion of pro‐inflammatory factors, we treated *Spalax* RS cells exposed to CM MDA‐MB‐231, with a chemical antagonist of IL1R. A chemical antagonist binds the region of IL1R/MyD88 interaction, inhibiting the positive feedback loop of IL1α/NF‐κB, which, in turn, leads to a decrease in the level of IL6 expression (IL6 was used as indicator of SASP activation/suppression). As shown in Figure 6d, an IL1R antagonist weakened the stimulating effect of MDA‐MB‐231 CM, reducing the expression of IL‐6 mRNA in *Spalax* RS cells by 50%. For better understanding of this experiment, we present a simplified diagram explaining the inhibitory effect of an IL1R antagonist on SASP (IL6) expression in *Spalax* fibroblasts caused by inflammatory factors of MDA‐MB‐231 CM (Figure 6e).

These data suggest that inhibition of IL1α expression and its poor availability on the plasma membrane are involved in the suppression of SASP in senescent *Spalax* cells. In addition, the transcription factor GATA4, the upstream regulator of NF‐κB and IL1α (Kang et al., 2015), which had a relatively weak signal in early‐passage fibroblasts of human and *Spalax* cells (Figure S10), raised three times in human senescent fibroblasts, but moderately increased one‐half‐fold in RS *Spalax* cells. However, GATA4 in RS *Spalax* cells was significantly amplified under the influence of MDA‐MB‐231 CM, probably due to inhibition of GATA4 autophagic degradation (Figure S10B,C).

### 2.9 In vivo evaluation of expression of genes related to SASP in young and aging *Spalax* individuals

**Figure 7:**
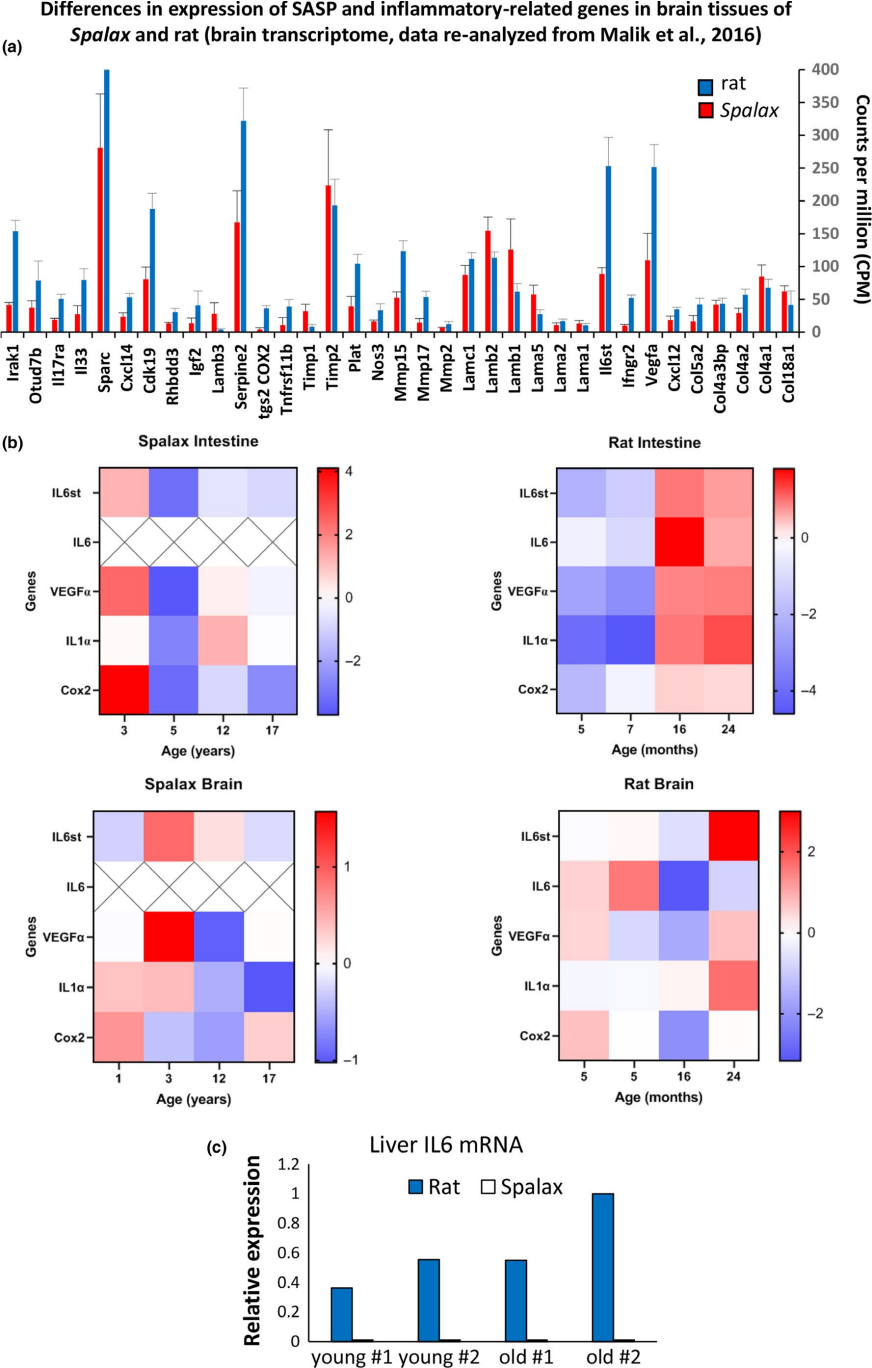
Evaluation of SASP gene expression *in vivo*. (a) Differences in expression of genes related to SASP and inflammatory response in *Spalax* and rats (re‐analyzed data of brain transcriptome; Malik et al., 2016); (b) mRNA expression of SASP genes *in* *vivo*: Frozen brain, intestine, and liver tissues of young and old *Spalax* and rat individuals were used for RNA isolation (details are listed in Table [Link], [Link]). Totally, 12 tissue samples of *Spalax* and 12 tissue samples of rats (three young/adult and two old individuals from each species) were used in the experiments. A heatmap was generated based on quantitative analysis of RT–qPCR, and it represents relative gene expression (log_2_ fold changes). Rows represent genes; the columns represent individuals. "X"—undetectable level. Data of individual RT–qPCR in bar graphs are presented in Figures S11 and S12 ; (c) relative expression of IL6 mRNA in liver tissues of young and old rat and *Spalax* individuals. Rat: young—5 and 7 months old (#1 and #2); old—16 and 24 months old (#1 and #2). *Spalax*: young—3 and 5 years old (#1 and #2); and old—12 and 17 years old (#1 and #2)

To extend our investigation beyond cultured cells, we addressed the question whether old *Spalax* individuals have a reduced expression level of inflammatory mediators. For this purpose, we first re‐analyzed data of *Spalax* brain transcriptome that we previously published (Malik et al., 2016). In this work, we compared whole‐brain transcript abundance profile of 6 *Spalax* (from 1–17 years old) and 6 rat individuals (from 5 to 24 months old). In Figure 7a, we present average expression levels of genes relating to SASP and individual genes associated with inflammation and NF‐κB regulation [Malik et al., 2016; Table S5 therein]. SASP genes that are not presented in Figure 7a (IL6, IL8, IL1A, GROα, SerpinB2, IGFBPs 5 and 7, ICAM, SDF) were below the level of detection or had a very poor transcription in the brain of both species. As shown, most genes related to the SASP and inflammation are upregulated in rats compared with *Spalax*. In rat brain, expression of IL6st, VEGFα, MMP15, MMP17, NOS3, CXCL12, CXCL14, and Cox2 was more than 2 times higher than that of *Spalax*. In addition, the expression of interleukin‐1 receptor‐associated kinase 1 (IRAK1), which plays a central role in the initiation of the inflammatory response during IL1R or TLR stimulation, is significantly reduced in *Spalax*'s brain. An increased expression of IRAK1 is associated with Behcet's syndrome, which is characterized by multiple inflammation in the body (Sun, Yang, Yang, & Ye, 2018). Expression of the SPARC gene (osteonectin), which is a MMP‐stimulating factor (Lund, Giachelli, & Scatena, 2009), is also inhibited in *Spalax* brain tissues compared to rats. Among the SASP and pro‐inflammatory genes that were found to be significantly upregulated in *Spalax* *vs*. rats were TIMP1 (tissue inhibitor of metalloproteinases) and a set of genes coding laminins α and β (Lamb 1, 2, and 3 and Lama 5), all of which belong to the extracellular matrix (ECM) group (Malik et al., 2016). As was demonstrated in another *Spalax* brain transcriptome microarray study by our group, ECM‐related cluster of genes was "overrepresented among hypoxia responsive genes, and functionally‐related with hypoxia‐induced angiogenesis" (Malik et al., 2012).

To test whether SASP genes are differently expressed in aging *Spalax*, we selected IL6, IL1α, and Cox2, which were used in our *in vitro* investigations, and added two other known SASP genes, VEGF and IL6st (signal transducer interleukin‐6), with a high expression rate in *Spalax* and rat brain tissues (Figure 7a). RNA was extracted from frozen tissues of the brain, intestine, and liver of young and old *Spalax* and rat individuals (detailed characteristics of animals that were used for obtaining tissue samples are presented in Table [Link], [Link]). Relative expression of SASP genes in the intestine and brain of old and young *Spalax* and rats according to qRT–PCR data is presented in Figure 7b (heatmap) and in Figures S11 and S12. As demonstrated, in the intestine of *Spalax*, IL‐6 expression was not detected in all animals regardless of age. In the intestines of rats, IL6 increased in old animals (16 and 24 months) *vs*. young (5 and 7 months). Similarly, IL6st, VEGFα, IL1α, and Cox2 were activated in old rats. In contrast, IL6st, VEGFα, and Cox2 were reduced in the intestines of old *Spalax* individuals (12 and 17 years old) compared to the youngest ones (3 years old). IL1α expression was equally low in the intestines of the youngest and oldest *Spalax* individuals. In the brain of *Spalax*, IL1α gradually decreased from young to old and, conversely, gradually increased with age in the brain of a rat. VEGFα and Cox2 also showed a downward trend in brain of older‐age *Spalax*. As in the intestine, the expression of IL6 mRNA in *Spalax's* brain was not detected. In addition, we tested the expression of IL6 in *Spalax* and rat liver tissues, and as shown in Figure 7c, IL6 mRNAs in *Spalax* samples were at a very low level of expression, while in rat liver, the expression of the IL6 gene increased with age.

## 3 DISCUSSION

In this study, we demonstrated a unique feature of cellular senescence in the fibroblasts of *Spalax*, a long‐lived and cancer‐resistant subterranean rodent. *Spalax* senescent fibroblasts were characterized by proliferative arrest, SA‐β‐Gal‐positive staining, and increased expression of p21, p16, and p53; nevertheless, signs of cellular senescence in *Spalax* were not accompanied by the secretion of canonical SASP inflammatory factors (IL6, IL8, IL1α, SerpinB2, GROα, ICAM‐1) following RS and EIS. In contrast, the mRNA expression level of the anti‐inflammatory cytokine IL10 increased in *Spalax* senescent fibroblasts but was undetectable in mouse and human cells. Evaluation of gene expression of individual genes relating to SASP in intestine and brain showed a downward trend in older‐age *Spalax* compared to young individuals.

Replicative senescence is usually associated with telomere shortening, but it can be triggered by other stress stimuli (Kuilman, Michaloglou, Mooi, & Peeper, 2010); of special interest is the finding that irreparable telomeric damage may trigger RS, irrespective of telomere length (Fumagalli et al., 2012). Several types of cells undergo senescence before telomere erosion can be recognized as DNA damage. This type of premature senescence termed stress‐induced premature senescence (SIPS) was observed in mouse embryonic fibroblasts (MEFs). MEFs undergo senescence before any considerable signs of telomere attrition; accumulated senescent MEFs amass DDR foci and show activation of p53 and SASP (Calado & Dumitriu, 2013). Similar to those of the mouse, *Spalax* fibroblasts cultured in standard conditions after a limited number of population doublings attain a flat and enlarged morphology and become positive for senescence markers. In this report, we did not consider the relationship between replicative senescence in *Spalax* and telomere shortening in culture; however, an *in vivo* study has shown that in *Spalax* tissues telomeres shorten with age (Adwan Shekhidem et al., 2019), indicating that rather telomere maintenance and integrity plays a critical role the suppression of SASP. The main goal of the current study was determining whether cellular senescence in *Spalax* is accompanied by the development of canonical SASP, regardless of the type of senescence (replicative, SIPS, or etoposide‐induced). Human fibroblasts were used for comparison with *Spalax* not because of human longevity, but rather due to the well‐characterized SASP in these cells; mouse fibroblasts were taken as another rodent cell model.

Since SASP is a direct consequence of DNA damage, we used low concentration of etoposide, a DNA‐damaging agent, to induce premature senescence and DDR, which is capable of inducing senescence but not apoptosis (Tamamori‐Adachi et al., 2018). In response to etoposide, human and mouse cells demonstrated increased γH2AX foci number/nucleus, whereas in *Spalax* cells the level of DSBs remained low as in the control. Nevertheless, *Spalax* fibroblasts, similarly to human and mouse cells, stopped dividing and underwent irreversible proliferative arrest; moreover, they showed a positive SA‐β‐Gal staining and increased gene expression of p21, p16, and p53, the important markers of senescence. Recently, we demonstrated that *Spalax* skin fibroblasts in culture resist several types of genotoxic insult (H_2_O_2_, UV‐C radiation, etoposide in high doses) and exhibit an enhanced DNA repair capacity compared with *Rattus* fibroblasts, evaluated by γH2AX immunofluorescence, comet assay and host cell reactivation assay (Domankevich et al., 2018). In addition, our previous studies demonstrated significant enrichment of DNA replication and Fanconi anemia pathways in *Spalax* brain, muscle, and liver transcriptomes (Altwasser et al., 2019; Malik et al., 2016; Schmidt et al., 2017), that contribute to DNA stability during replicative and genotoxic stress. Applying to our experimental conditions, we confirmed that the lack of accumulation of DNA damage in *Spalax* fibroblasts exposed to etoposide is due to the high efficiency of DNA repair (Figure 4). Thus, the DNA damage that etoposide induced in *Spalax* fibroblasts was repaired before it became persistent and capable of inducing SASP factors; however, senescence development had already initiated, and the disappearance of γH2AX due to DNA repair apparently could not reverse the process. In consistence with our findings, etoposide‐induced DSBs in HT‐29 human colon cancer cells (Schonn, Hennesen, & Dartsch, 2010) were quickly fixed long before the cancer cells underwent apoptosis, but the disappearance of DSBs did not abolish cell death, even after etoposide withdrawal.

In another cancer‐resistant subterranean mammal, the naked mole rat (NMR), fibroblasts treated with intensive γ‐irradiation underwent senescence accompanied by increased SASP gene expression (Zhao et al., 2018), and the authors concluded that the SASP response is conserved in senescent NMR cells; however, one would assume similar strategies convergently evolved in these two long‐lived mammals. The stress inducers and their intensity largely differ from those of the present study; moreover, the study on NMR cells did not include replicative senescence based on serial passaging of cells, which makes it difficult to compare the two studies. Nevertheless, a close look at the data presented therein shows extremely mild upregulation of important SASP factors, such as IL6, HGF, and ICAM in NMR cells compared with mouse cells, despite the severity of the applied stress; suggesting that senescence in NMR cells may have shown similarity with *Spalax* cells, specifically, in containing SASP inhibitory mechanisms. It is noteworthy that severe stress induced by MDA‐MB‐231 CM in the present study caused an inflammatory response in *Spalax* cells (discussed below), suggesting that severe artificial stress may mask the results and distract attention from the physiological strategies employed by these mammals.

Using different approaches, we provide convincing evidence that RS and EIS in *Spalax* fibroblasts are not associated with SASP in its canonical mode. Among the SASP regulators that stimulate the inflammatory response, the transcription factor NF‐κB plays a key role (Chien et al., 2011). Its activation via phosphorylation of the p65 subunit and its nuclear translocation are necessary events for triggering secretion of pro‐inflammatory factors via this pathway, including IL6 and IL8. *Spalax* senescent fibroblasts, like those of human and mice, demonstrated increased p65 phosphorylation (Ser^536^) compared with young cells, but in *Spalax* cells, this was apparently insufficient for inducing p65 translocation into the nuclei. It is noteworthy that the stimulation of inflammatory signaling in *Spalax* RS cells by treatment with MDA‐MB‐231 CM resulted in strong nuclear localization of p65, with subsequent activation of mRNA transcription IL6, IL8, and other SASP factors. A number of investigations have provided evidence that growth factors and inflammatory cytokines are capable of triggering NF‐κB signaling and inducing an inflammatory response in cancerous and noncancerous cells (Aivaliotis et al., 2012; Hubackova et al., 2012). Growth factors IL1α, IL1β, IL6, IL8, and tumor necrosis factor‐α (TNFα) or a combination thereof can activate genomic instability, single‐strand breaks, or DSBs, which in turn cause a persistent DDR and inflammatory response. Human breast cancer MDA‐MB‐231 is an extremely aggressive metastatic cell line producing vast amounts of growth factors, among which IL1β was identified as a potent stimulator of NF‐κB. Silencing of IL1β in MDA‐MB‐231 cells with shRNA resulted in the loss of the ability of their CM to activate NF‐κB in mesenchymal stem cells (Escobar et al., 2015). IL1β induces inflammatory genes through the IL1 receptor (IL1R), which, in turn, affects IL1R‐linked upstream regulators of NF‐κB. It can be assumed that either IL1β or a cocktail of cytokines contained in MDA‐MB‐231 CM causes a persistent activation of NF‐κB in *Spalax* fibroblasts, leading to translocation of NF‐κB to the nuclei and production of inflammatory mediators. The cytokines and growth factors in the inflammatory milieu may stimulate replication stress with subsequent formation of DSBs and activation of DDR (Aivaliotis et al., 2012). Thus, it appears that in conditions where the stability of DNA in *Spalax* cells is compromised by the continuous activation of surface inflammatory receptors (IL1R/Toll‐like family) and the production of internal cytokines enhances and supports inflammatory responses in *Spalax* cells undergoing replicative arrest, DNA repair mechanisms in *Spalax* can no longer act as a deterrent to producing SASP.

IL1α is an important regulator of the IL6/IL8 cytokine network (Orjalo et al., 2009). Both IL1α and IL1β bind the same IL1R receptor and induce similar biological effects associated with inflammation and immunity (Apte et al., 2006). Unlike IL1β, which acts as a secreted protein, IL1α stimulates an inflammatory response, mainly as a surface membrane‐bound protein, which is usually abundant in aging cells; IL1α binds the IL1R receptor in a juxtacrine manner, initiating a signaling cascade that blocks intracellular NF‐κB inhibitors to allow nuclear translocation of NF‐κB and subsequent activation of SASP (Freund, Orjalo, Desprez, & Campisi, 2010). We have demonstrated that an IL1R antagonist weakens the stimulating effect of CM MDA‐MB‐231 on the expression of IL‐6 mRNA in *Spalax* RS cells. Although further research is needed, this finding suggests that the positive feedback loop of IL1α/NF‐κB appears to be impaired in *Spalax*, and this is one of the reasons why inflammation is inhibited in *Spalax* aging cells. In addition, transcription factor GATA4, the upstream NF‐κB activator (Kang et al., 2015), appears to retain, at least partially, the ability of autophagic degradation in aging *Spalax* cells, thus inhibiting NF‐κB. But exposure to cancer cell CM led to a significant accumulation of GATA4 in RS Spalax fibroblasts, which probably affects SASP. Regulation of inflammatory response in *Spalax* by GATA4 needs to be further investigated both *in vivo* and *in vitro*.

Noteworthy is the finding that the observed suppression of pro‐inflammatory signals was not limited only to *Spalax* cultured cells. A substantial amount of *in vivo* results obtained in the present study and based on re‐analyzing previously published works of our group show a clear suppression, and in some cases, a downregulation of SASP gene transcription during aging. These results support the notion that suppressed SASP is a general *Spalax* evolutionary strategy that supports its outstanding longevity and cancer resistance.

In conclusion, we propose that suppression of SASP in senescent *Spalax* cells, as well as inhibition of inflammation in aging *Spalax*, can support a nonpermissive microenvironment for cancer progression, as well as the suppression of sterile inflammation, which is the cause of most aging‐related pathologies. The understanding of naturally evolved, fine‐tuned mechanisms for suppressing the inflammatory response in *Spalax* senescent cells during its millions of years of evolution in its underground stressful environment could pinpoint new avenues for antiaging therapeutic strategies based on the "uncoupling" of the senescence phenotype and the inflammatory component that accompanies it in all other species studied to date.

## 4 EXPERIMENTAL PROCEDURES

### 4.1 Animals, cell culture, and treatments

*Spalax* were captured in the field as they cannot be bred in laboratory conditions. Mice (*Mus* *musculus*) C57BL/6 and rats (*Rattus norvegicus*) were purchased from Envigo. Fibroblasts were isolated from newborns of mice (at the age of 2–7 days) and *Spalax *(at an estimated age of 2–14 days) as described in Glaysher and Cree (2011). Human foreskin fibroblasts were obtained from ATCC. Tissues were harvested and flash‐frozen from sacrificed animals used in previous work (Malik et al., 2016). All animal experiments were approved by the Ethics Committee of the University of Haifa [Reference #316/14 for cell isolation; and Reference # 193/10 for *in vivo* experiments].

Fibroblasts were grown in DMEM–F12 medium (supplemented with 10% FBS, L‐glutamine (2mM), and penicillin–streptomycin (100 µ/ml, 0.1 mg/ml, respectively)) in a standard CO_2_ incubator. Growth media and supplements were purchased from Biological Industries (Beit HaEmek, Israel). Fibroblasts were subjected to serial passages to achieve replicative senescence. The cells were defined as having a senescent phenotype when most of the cells acquired an enlarged, flattened morphology, cell division decreased below 25% of the total population, and the cells exhibited positive SA**‐**β‐Gal staining.

Population doubling levels were assessed as follows: 2.5 × 10^4^ cells were plated in six‐well plates. After 24, 48, 72, and 96 hr, cells were trypsinized and counted in triplicate.

#### 4.1.1 Induction of senescence by etoposide

To induce genotoxic stress, fibroblasts were treated with etoposide at a concentration of 1 µg/ml for 5 days; thereafter, cells were washed by PBS and incubated for an additional 3 days without etoposide. A similar treatment regimen with low etoposide concentrations was used in previous reports to induce senescence and SASP in different normal and cancer cells (Gu & Kitamura, 2012; Leontieva & Blagosklonny, 2010; Yosef et al., 2017).

##### Treatment with MDA‐MB‐231 CM and lipopolysaccharide (LPS)

To generate CM, MDA‐MB‐231 cells were cultured in RPMI medium supplemented with 10% FBS, until they reached 70% confluence (usually 48 hr). Complete supernatant was collected (centrifuged at 120g for 5 min at RT) to eradicate any cell debris.

To induce the inflammatory response in *Spalax* fibroblasts, cells were treated with MDA‐MB‐231 CM (diluted 1:1 with fresh medium) for 24 hr. After the treatments, cells were washed and collected for analysis (Western blot and qPCR) or fixed and stained for γH2AX, IL1α, NF‐κB‐p65, GATA4, and membrane tracker. To inhibit IL1 pathway, the chemical antagonist of IL1R (CAS No. 566914–00–9; Cayman Chemical, Michigan, USA) was added 1 hr prior to the addition of MB‐231 CM to *Spalax* RS cells and left with CM for 24 hr. After the treatment, the levels of IL6 mRNA were analyzed (treated with CM + IL1R antagonist *vs*. CM alone). LPS 1 µg/ml was added to *Spalax* RS cells for 1 hr; thereafter, untreated and LPS‐treated cells were fixed and immunostained for IL1α and NF‐κB–p65.

### 4.2 EdU labeling for the detection of dividing cells

For EdU incorporation assay, EIS fibroblasts were seeded on glass coverslips (2.5 × 10^4^ cells/well in six‐well plates). After 48 hr, cells were fixed and labeled by using Click‐iT EdU Alexa Fluor 488 Imaging Kit (Thermo Fisher, Cat. No. C10637) according to the manufacturer's recommendations. DNA was counterstained with Hoechst 33,342 (5 µg/ml final concentration) and mounted in Antifade solution. Quantitative analysis was performed using *ImageJ* software. At least 250 cells from independent fields were scanned.

### 4.3 Statistics and reproducibility

Experiments were repeated at least three times, unless mentioned otherwise. Fibroblasts of *Spalax* and mice were isolated from three different individuals independently. The data are presented as mean ± *SD* or as representative data and biological replicates in the Supporting Information. The Mann–Whitney nonparametric test was applied to test the differences between groups; *p* < .05 was considered significant.

The following methods are described in Supporting Information (Appendix [Link], [Link]): SA‐β‐Gal staining, immunoblotting, immunofluorescence, flow cytometry, preparation of RNA and cDNA, quantitative real‐time polymerase chain reaction (RT–PCR), and enzyme‐linked immunosorbent assay (ELISA). Information about antibodies used for the Western blot and immunofluorescence is presented in Table [Link], [Link]. Primers used for quantitative real‐time PCR are presented in Table [Link], [Link]. Although mRNA expression may not necessarily reflect protein expression and secretion, it was the method of choice in many of our experiments as it allows the design of species‐specific primers for accurate quantification and avoids possible difficulties anticipated in antibody‐based analyses for nonclassical organisms. Tissue samples and descriptions of animals from which these samples were obtained for RT–qPCR are presented in Table [Link], [Link].

## CONFLICT OF INTERESTS

None declared.

## AUTHOR CONTRIBUTIONS

I.M., A.O., and I.S. planned and designed the experiments; A.O. and M.D. carried out the experiments; A.O., I.M., I.S., and V.D. analyzed the results and wrote the manuscript; and I.M. and I.S. supervised the project.

## Data Availability

The authors confirm that data supporting the findings of this study are available within the article or its supporting information.
