# Supplementary figures and images for: Downregulation of the inflammatory network in senescent fibroblasts and aging tissues of the long‐lived and cancer‐resistant subterranean wild rodent, Spalax
Source: Aging Cell. 2019 Oct 11;19(1):e13045. doi: 10.1111/acel.13045 (PMC6974727; doi:10.1111/acel.13045)

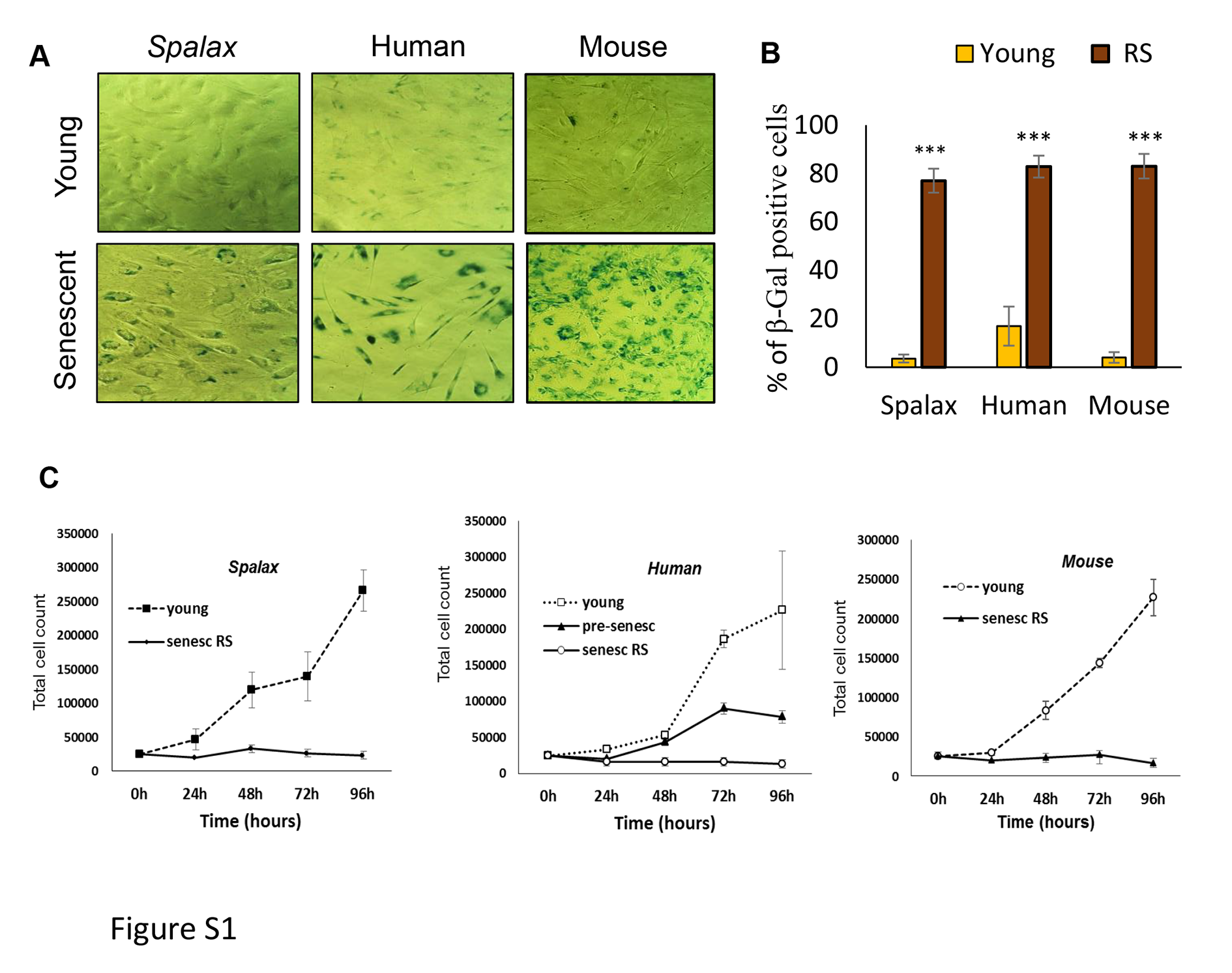

Supplement: Supplementary file 1 [file ACEL-19-e13045-s001.tif]

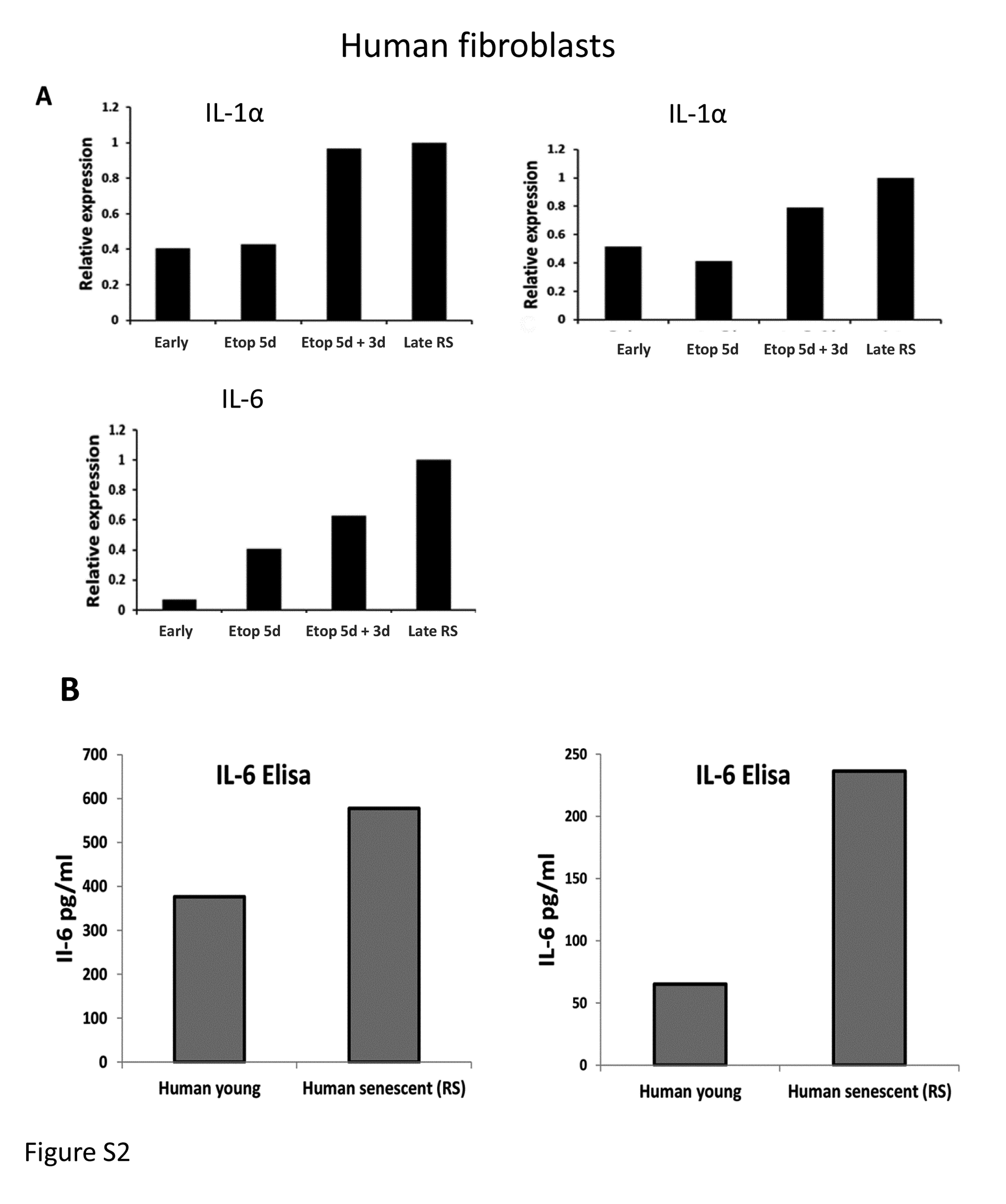

Supplement: Supplementary file 2 [file ACEL-19-e13045-s002.tif]

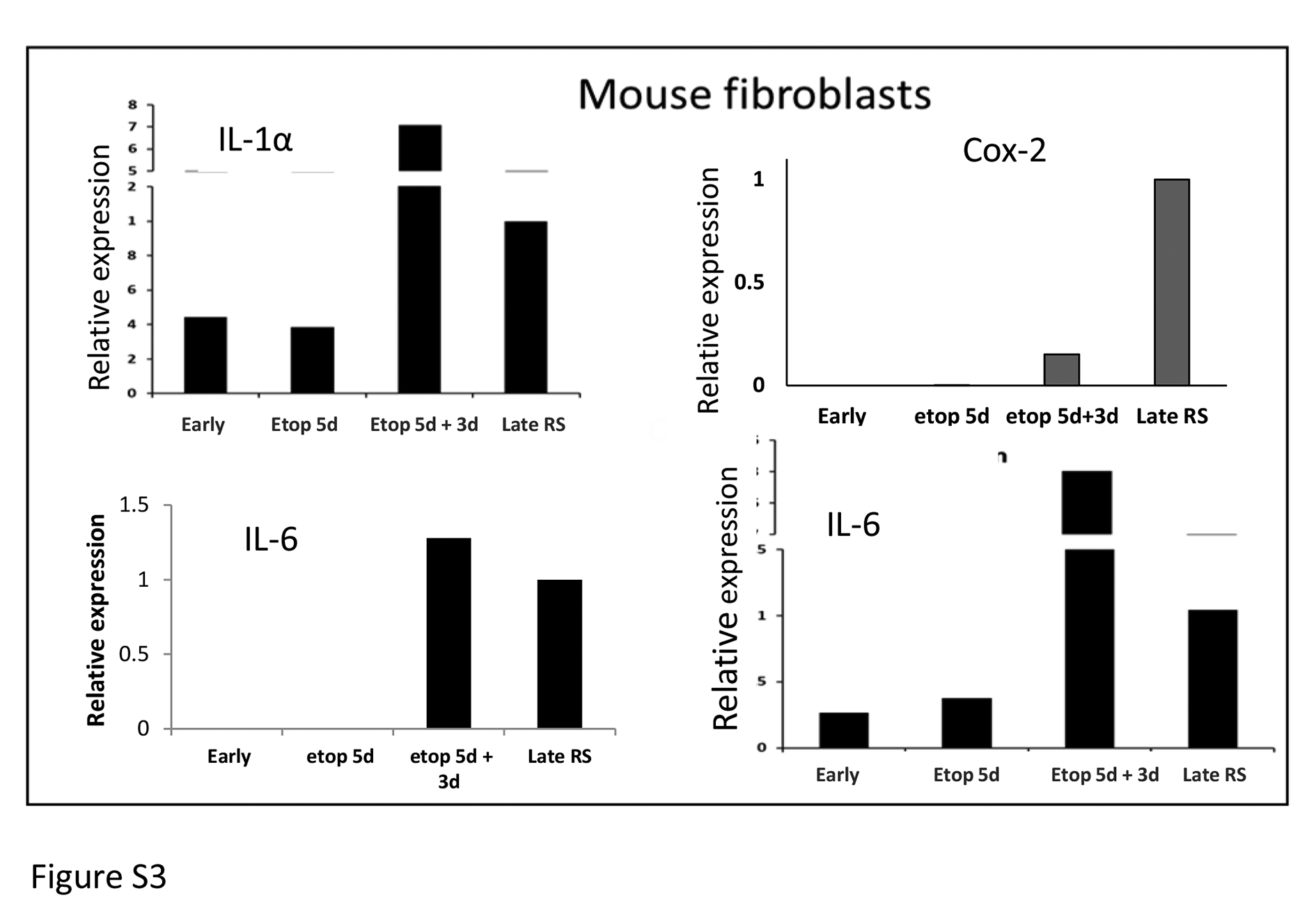

Supplement: Supplementary file 3 [file ACEL-19-e13045-s003.tif]

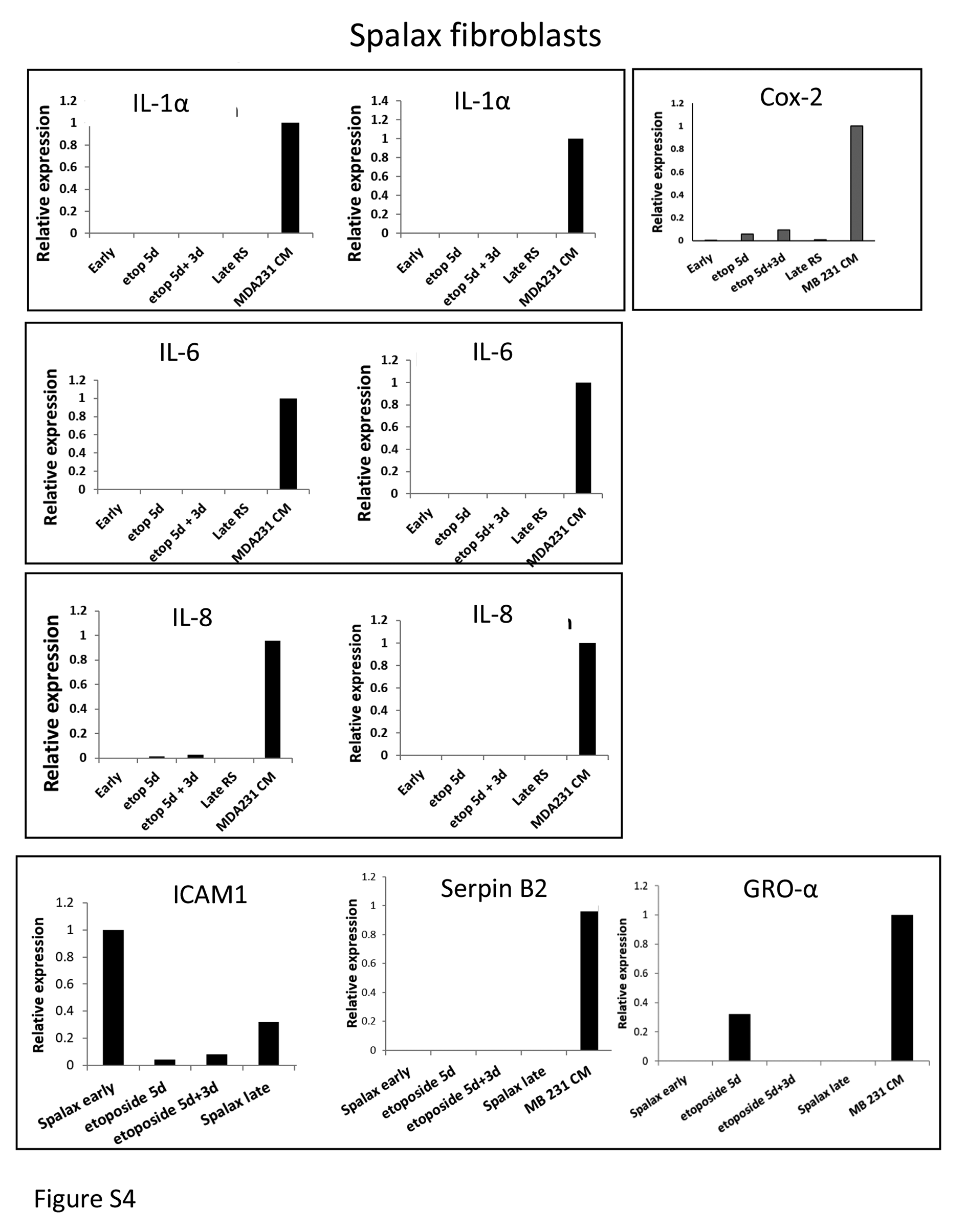

Supplement: Supplementary file 4 [file ACEL-19-e13045-s004.tif]

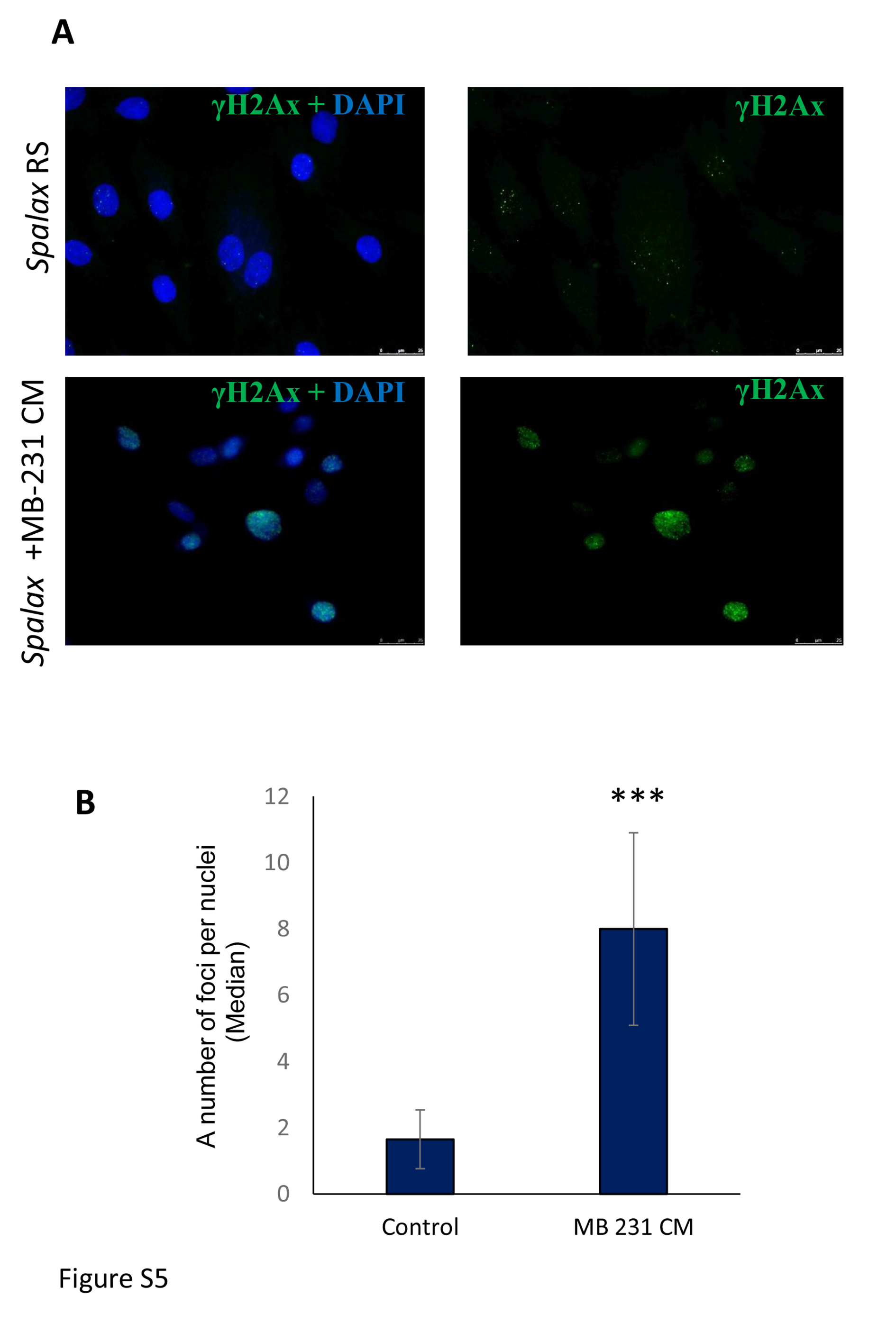

Supplement: Supplementary file 5 [file ACEL-19-e13045-s005.tif]

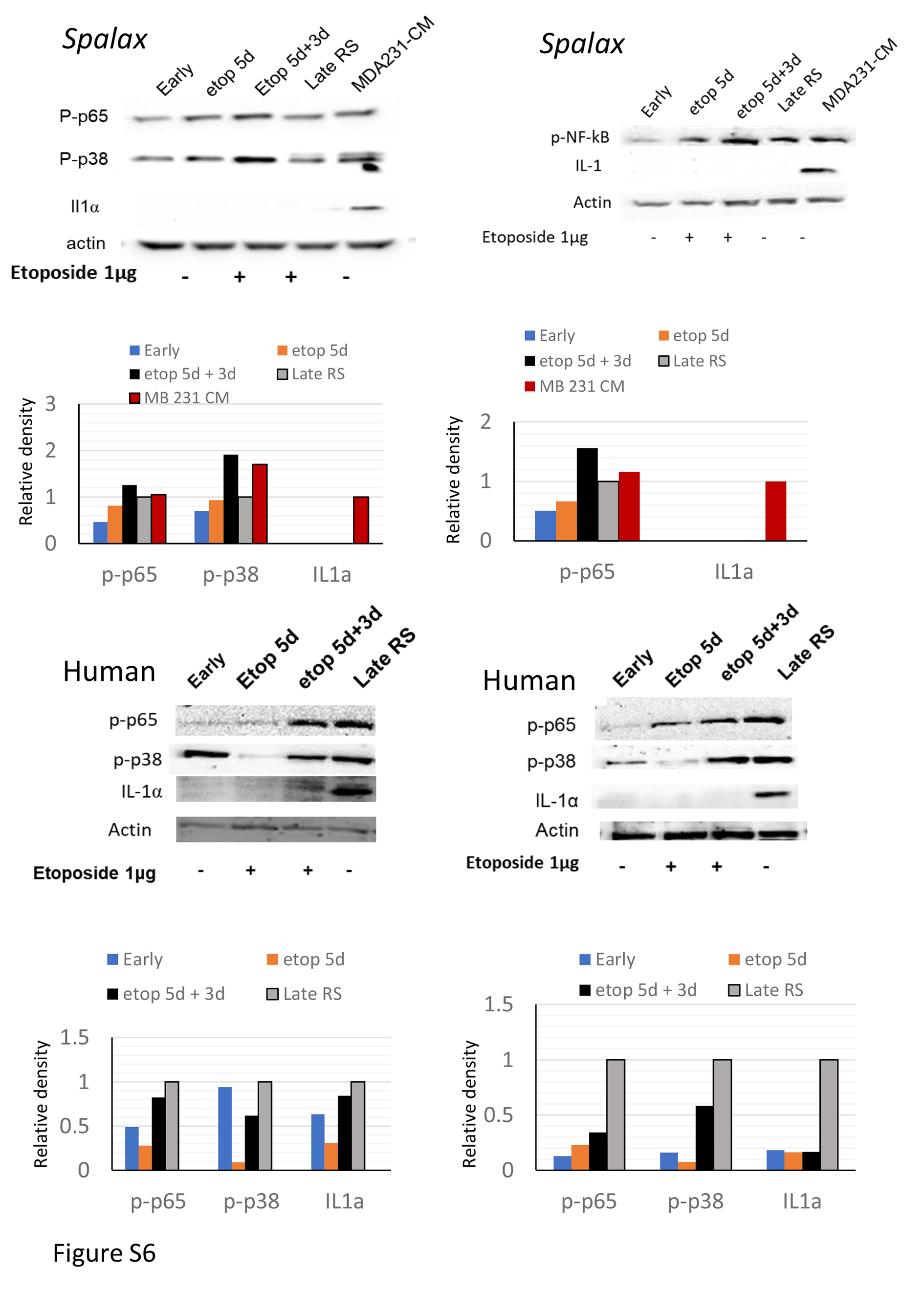

Supplement: Supplementary file 6 [file ACEL-19-e13045-s006.tif]

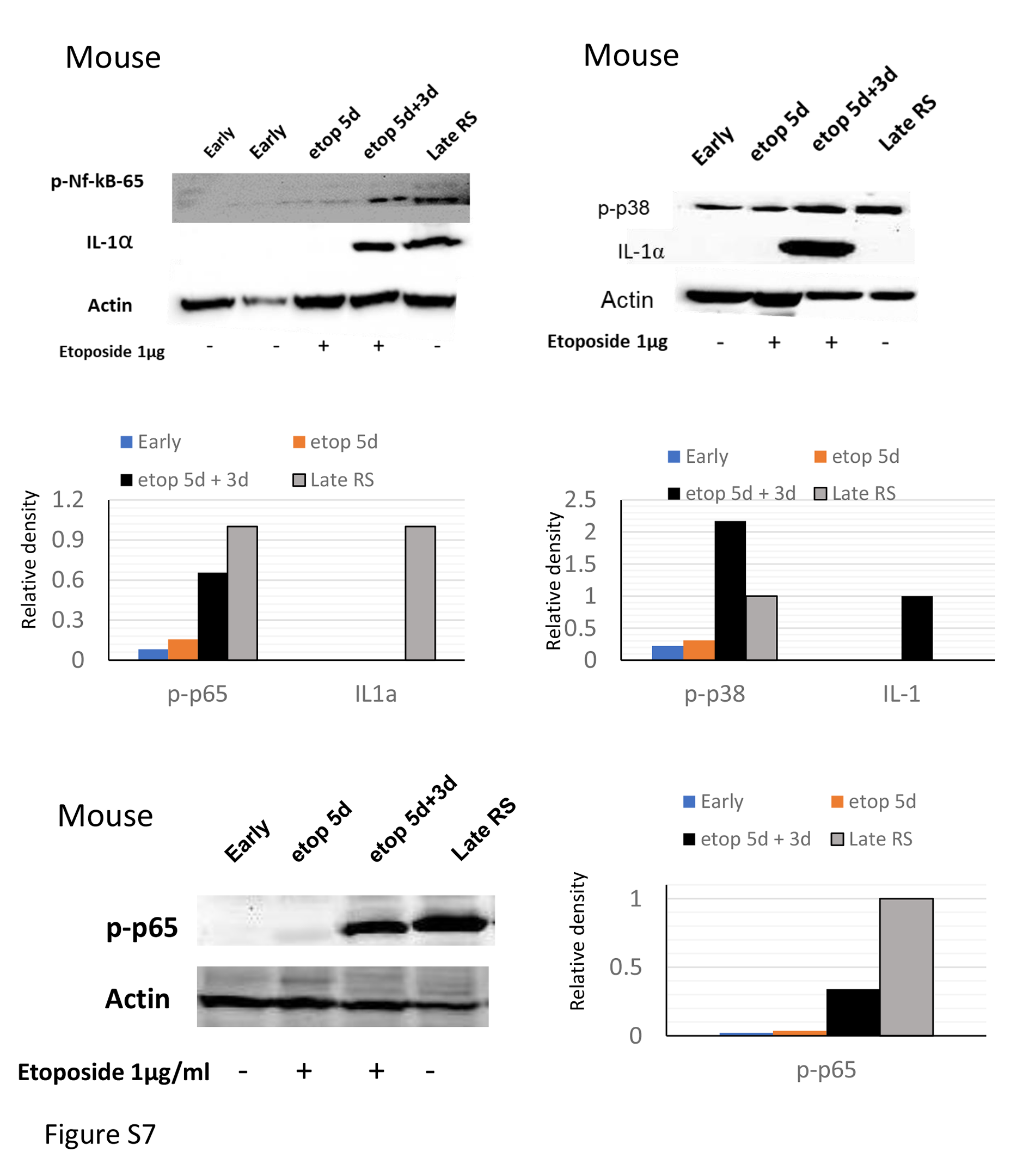

Supplement: Supplementary file 7 [file ACEL-19-e13045-s007.tif]

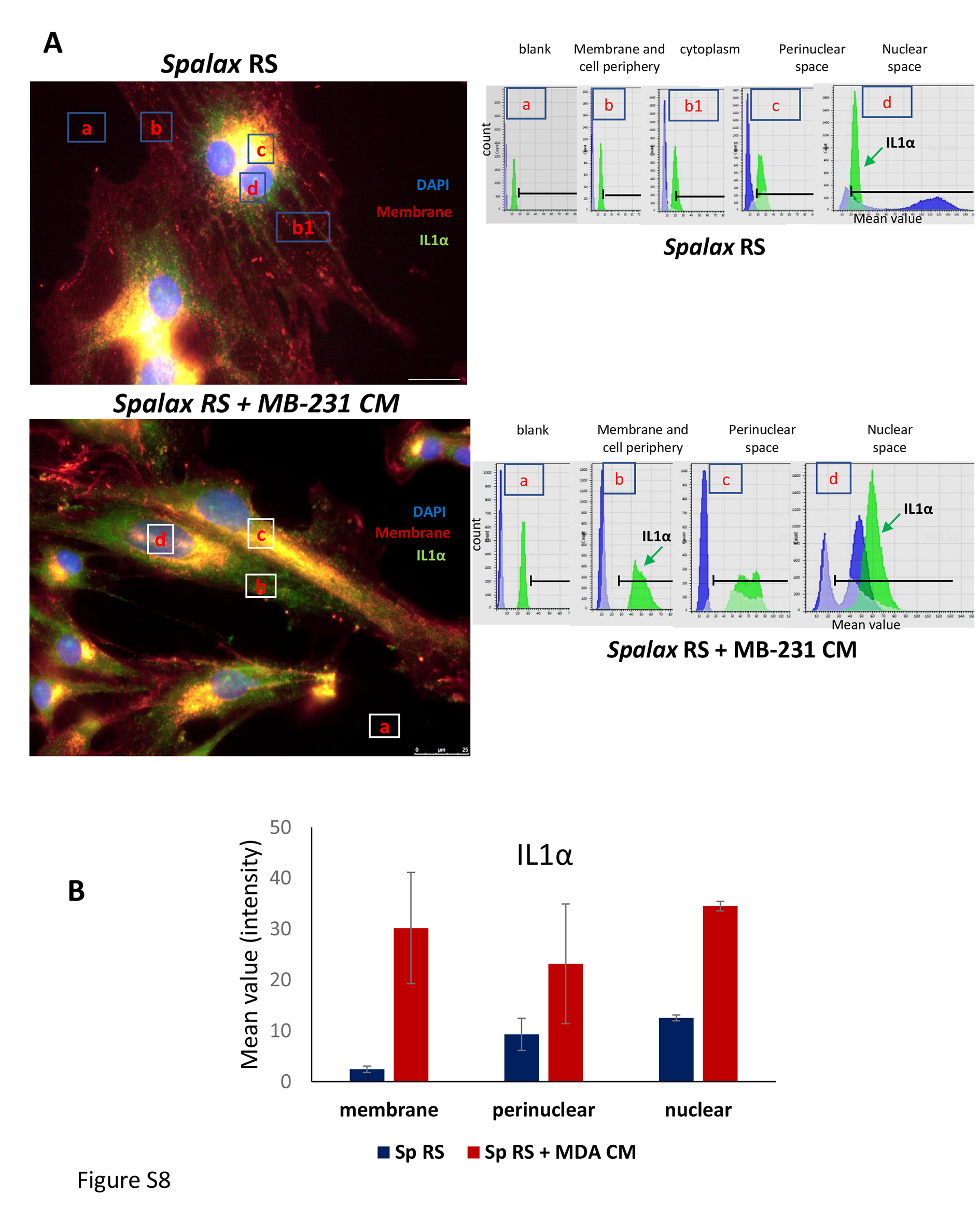

Supplement: Supplementary file 8 [file ACEL-19-e13045-s008.tif]

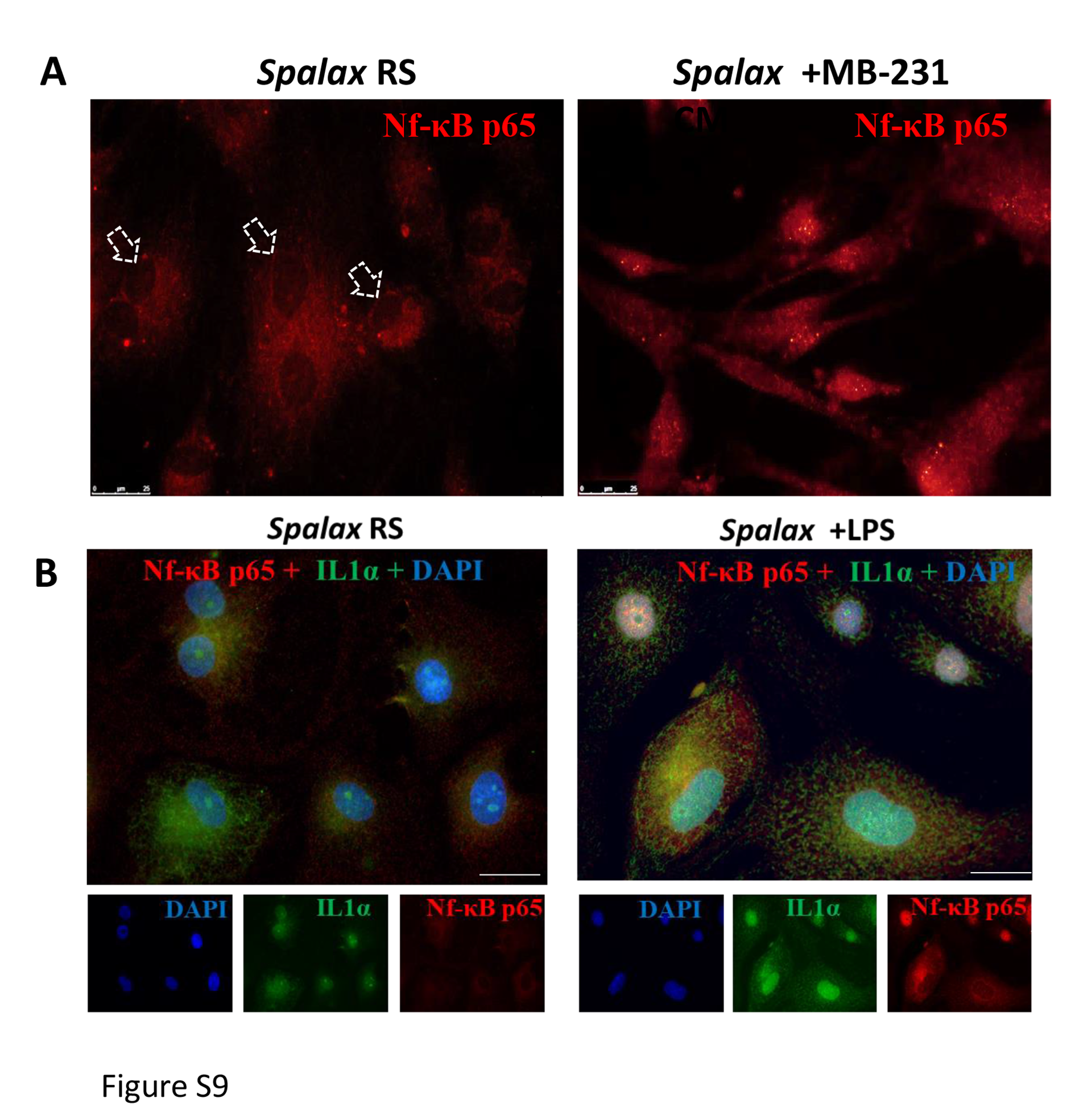

Supplement: Supplementary file 9 [file ACEL-19-e13045-s009.tif]

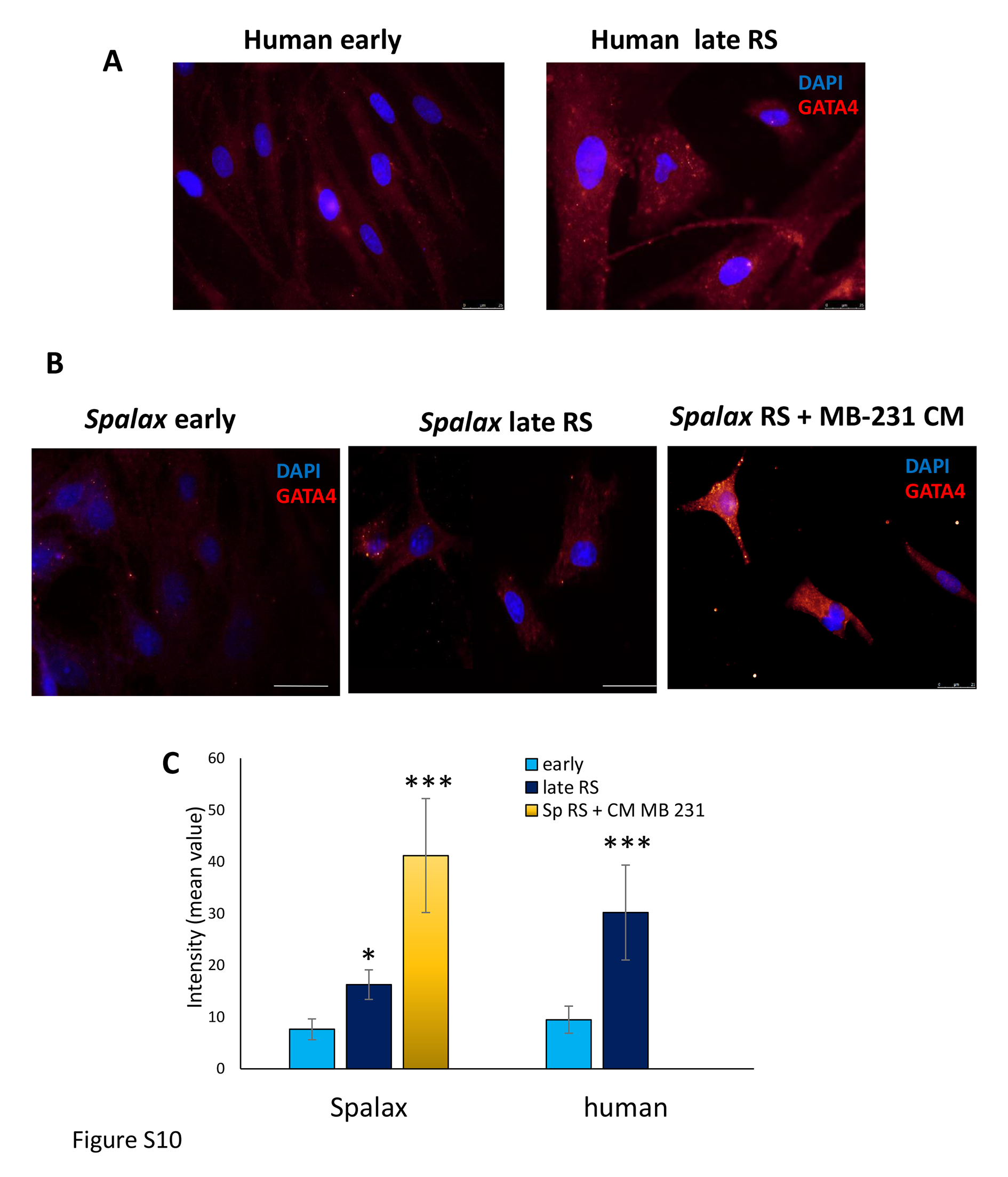

Supplement: Supplementary file 10 [file ACEL-19-e13045-s010.tif]

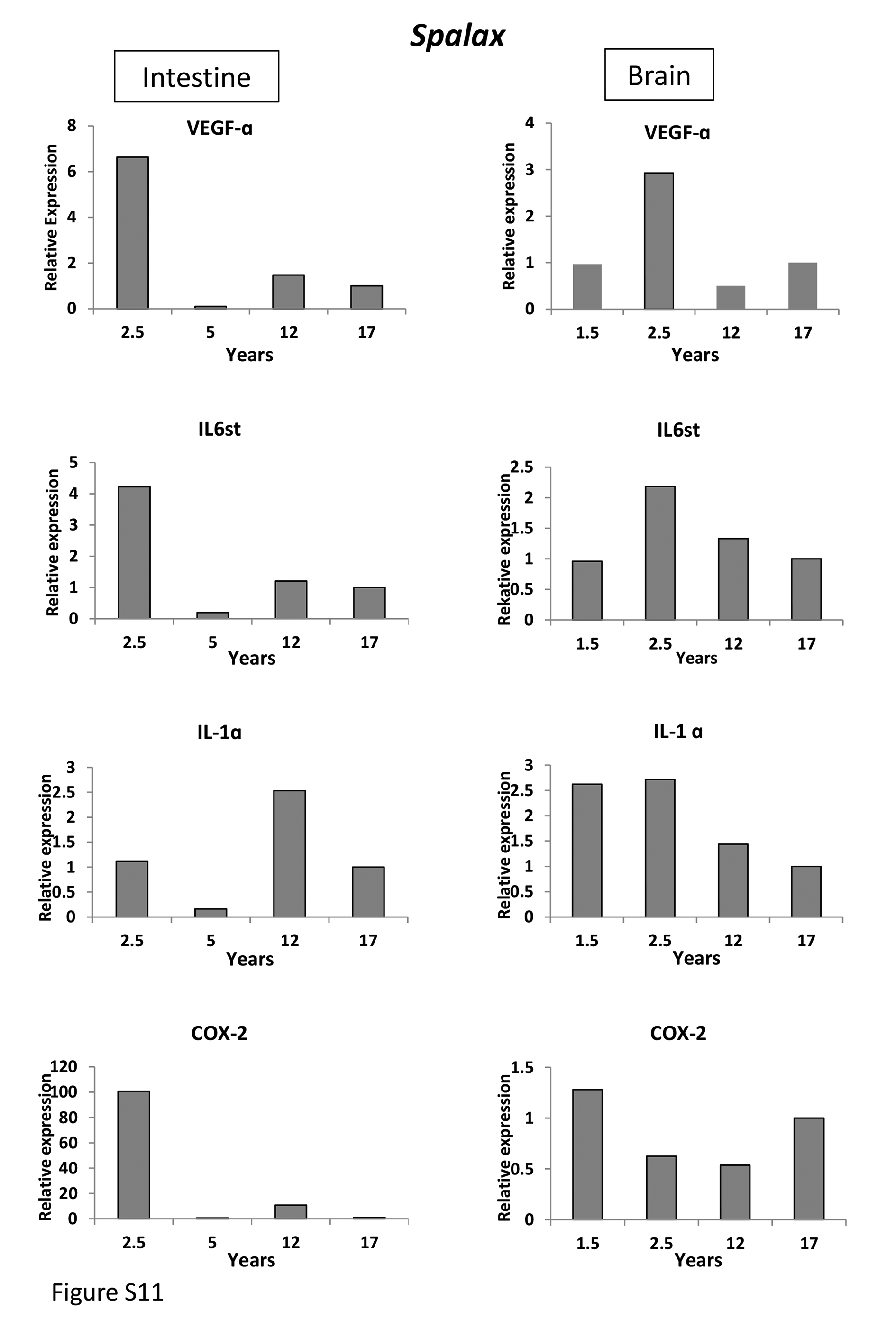

Supplement: Supplementary file 11 [file ACEL-19-e13045-s011.tif]

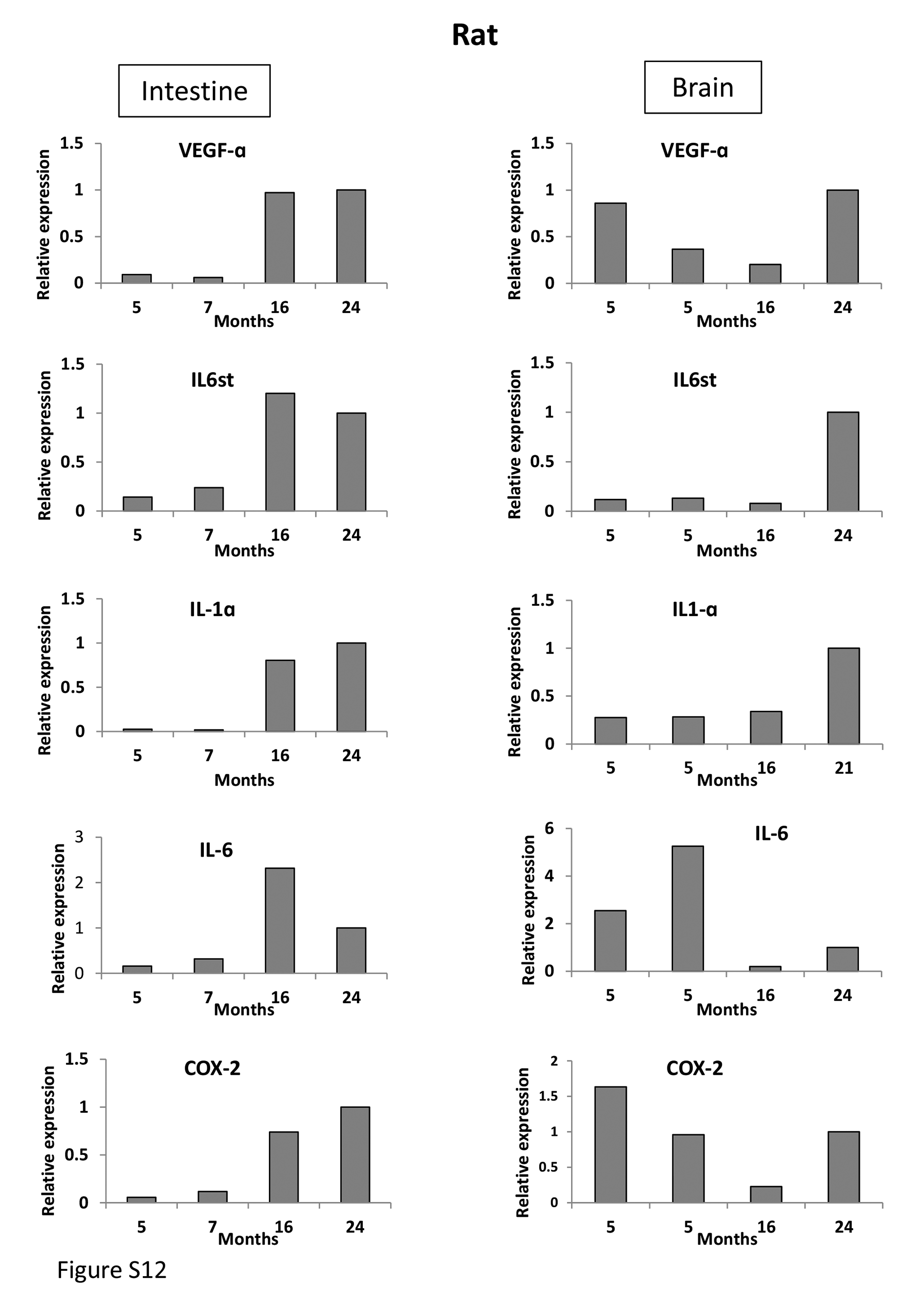

Supplement: Supplementary file 12 [file ACEL-19-e13045-s012.tif]

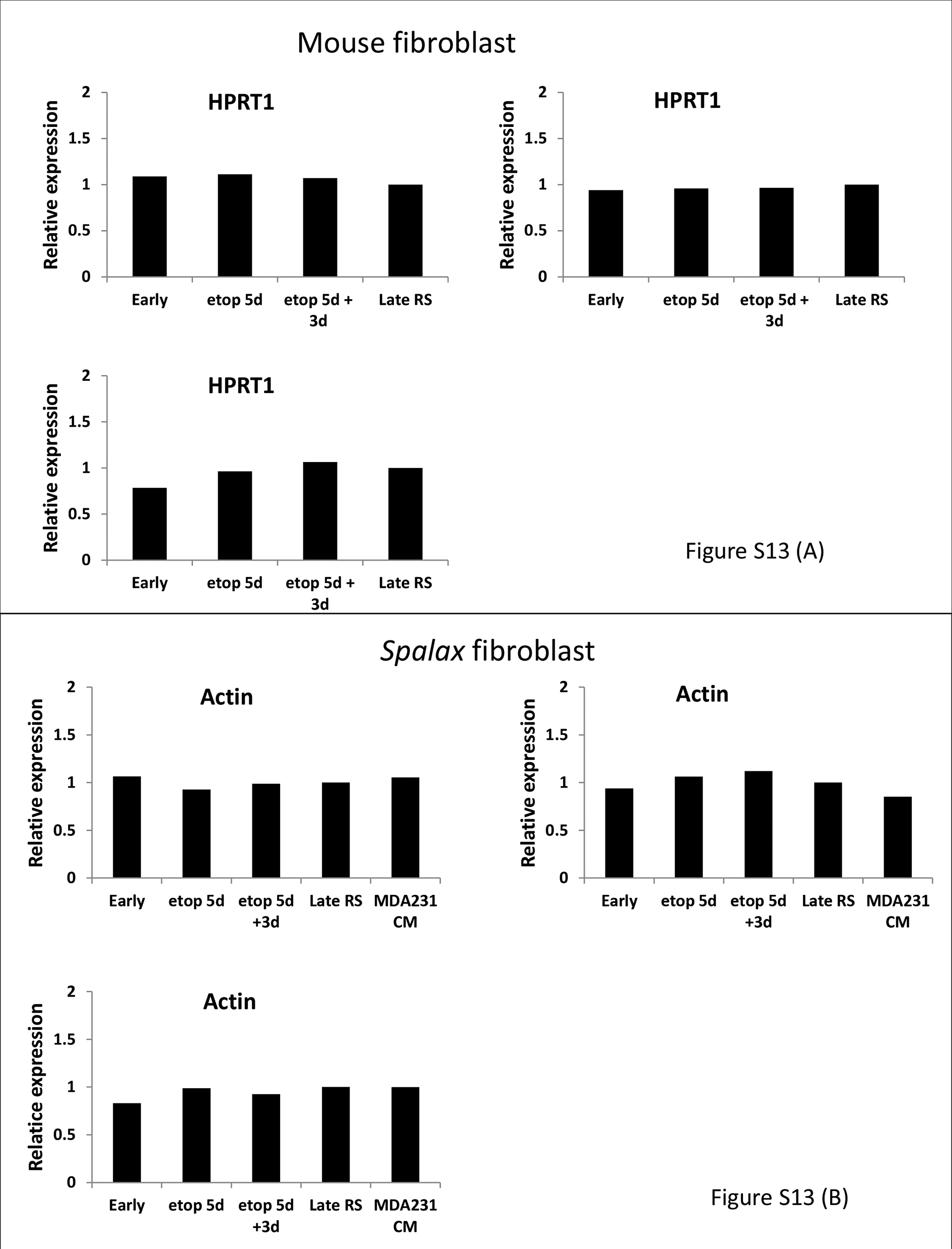

Supplement: Supplementary file 13 [file ACEL-19-e13045-s013.tif]

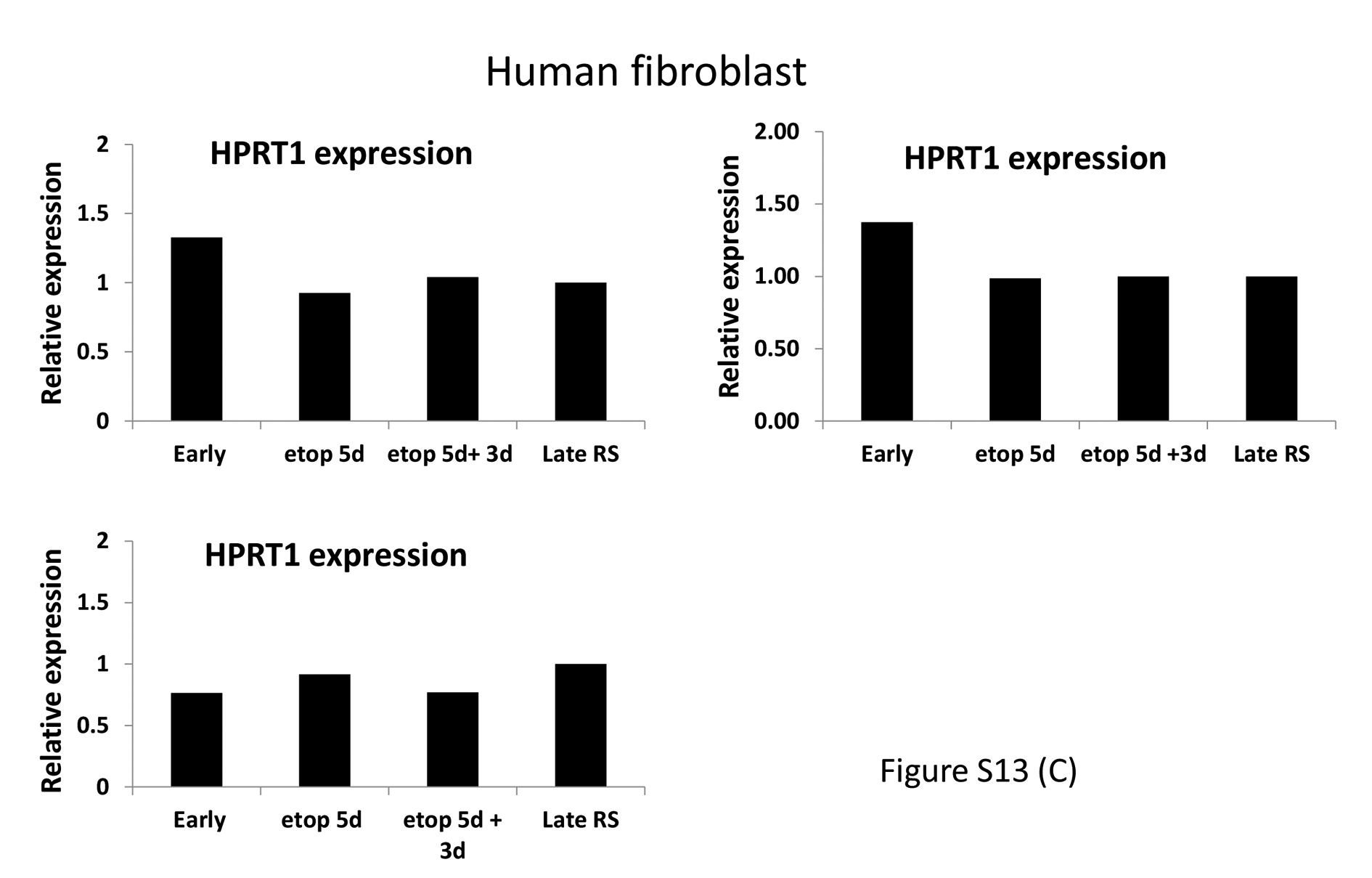

Supplement: Supplementary file 14 [file ACEL-19-e13045-s014.tif]
