# Supplementary material for: Downregulation of the inflammatory network in senescent fibroblasts and aging tissues of the long‐lived and cancer‐resistant subterranean wild rodent, Spalax
Source: Aging Cell. 2019 Oct 11;19(1):e13045. doi: 10.1111/acel.13045 (PMC6974727; doi:10.1111/acel.13045)
Supplement: Supplementary file 15 [file ACEL-19-e13045-s015.docx]

**Table S1. List of antibodies.**

| **Antigen Name** | **Dilution** | **Origin** | **Company** |
| --- | --- | --- | --- |
| **Primary antibodies Western blot analysis** | | | |
| Anti- phospho-NF-kB p65 (Ser536) | 1:1000 | Rabbit | Cell signaling |
| Anti -IL-1alpha -FITC | 1:1000 | Rabbit | Avivasysbio |
| Anti-phospho-p38 | 1:1000 | Rabbit | Abcam |
| Anti-actin | 1:300 | Goat | Santa Cruz |
| **Antibodies for fluorescent microscopy** | | | |
| Anti-gamma H2AX (phospho S139) | 1:700 | Rabbit | Abcam |
| Anti -IL-1alpha -FITC | 1:1000 | Rabbit | Avivasysbio |
| Anti- NF-kB p65 | 1:1000 | Rabbit | Cell signaling |
| Anti-GATA4 | 1:500 | Goat | Santa Cruz |
| **Secondary antibodies** |  |  |  |
| Anti-mouse CY3 | 1:300 | Donkey | Jackson ImmunoResearch  Lab. Inc. |
| Anti-rabbit CY2 | 1:300 | Donkey | Jackson ImmunoResearch  Lab. Inc. |
| Anti-rabbit Alexa 647 | 1:300 | Goat | Abcam |
| Anti-rabbit Alexa 488 | 1:300 | Goat | Abcam |
| Anti-rabbit HRP | 1:10000 | Goat | Jackson ImmunoResearch  Lab. Inc. |
| Anti-goat HRP | 1:10000 | Donkey | Jackson ImmunoResearch  Lab. Inc. |
