# Supplementary material for: Downregulation of the inflammatory network in senescent fibroblasts and aging tissues of the long‐lived and cancer‐resistant subterranean wild rodent, Spalax
Source: Aging Cell. 2019 Oct 11;19(1):e13045. doi: 10.1111/acel.13045 (PMC6974727; doi:10.1111/acel.13045)
Supplement: Supplementary file 16 [file ACEL-19-e13045-s016.docx]

**Table S2. Primers for RT-qPCR used in the *in vitro* and *in vivo* studies**

| **Primers** | **Forward** | **Reverse** |
| --- | --- | --- |
| **Spalax primers** | | |
| IL-10 | CCTGGCTCAGCACTGCTATG | TGGGTGTCCTCGCCTCTG |
| COX-2 | AGTGGCAAAGGCCTCCATT | AAGCGTTTGCGGTACTCATTG |
| Actin | GAGCATGGCATTGTCACCAA | CCACACGCAGCTCGTTGTAG |
| IL1-A | CGGCTGCCTATCCAGACCT | ATAGAGGGTAGGCCCCTTGC |
| IL-6 | CGGGAAACTTGCCTGTTGAA | ACTTGCCCTGGATGTACTCCA |
| IL-8 | CCTTTCTGCTCTCTGCAGCTG | TTGCACTGGCATCGAAGTTC |
| P21 | AACCTCTCAGGGCCGAAAAC | GGCGCTTGGAGTGGTAGAAA |
| P53 | GCACAAGCTCTTCTCCCCAA | TCACGCCCACGGATCTTAAG |
| GRO-ɑ | CCACTGCAGCCAGACAGAAGT | GGGCTTCAGGGTTAAGGCA |
| ICAM-1 | CCTTCCTACAACGAGGGACG | TCTAAGCCCAGGGTGAAGGTC |
| Serpb2 | GAGGGCACTATAGCAGCTGGC | CCACAAACTGTGGGCCTCC |
| IL6st | AACATGGCAGTGTGCATCAAA | TGCTCGCCACTAGGTTTCCT |
| VEGF-ɑ | GCTCAGAGCGGAGAAAGCAT | CGCGAGTCTGTGTTTTTGCA |
| P16 | ATCCCAACCGAGTCAACCG | AGTTCAGCAACCTGGGCACT |
| GATA4 | CACCAGACACCCCAATCTCG | CATGGCTCCACAGTTGACACA |
| **Human primers** | | |
| COX-2 | TTTTGGTGGAGAAGTGGGTTTT | CCCTTCACGTTATTGCAGATGA |
| IL-6 | TGTAGCCGCCCCACACA | CCGTCGAGGATGTACCGAAT |
| P53 | CCGAGTGGAAGGAAATTTGC | TAGGGCACCACCACACTATGTC |
| P21 | GCGGCAGACCAGCATGAC | GCGGATTAGGGCTTCCTCTT |
| HPRT1 | TGGGAGGCCATCACATTGTA | TGTAATCCAGCAGGTCAGCAAA |
| IL-1A | TCATCCTGAATGACGCCCTC | TTATGTAATGCAGCAGCCGTG |
| IL-10 | TGAAGACCCTCAGGCTGAGG | CACGGCCTTGCTCTTGTTTT |
| GRO-ɑ | GTGTGAACGTGAAGTCCCCC | GCTTTCCGCCCATTCTTGA |
| ICAM-1 | TGACGAAGCCAGAGGTCTCA | AGCGTCACCTTGGCTCTAGG |
| Serpb2 | GATTTTGCAGGCACAAGCTG | TGTGGATGCATTGATTGCAGA |
| P16 | GCGGAAGGTCCCTCAGACA | GATGATCTAAGTTTCCCGAGGTTTC |
| GATA4 | AAACCAGAAAACGGAAGCCC | GGAAGGCTCTCACTGCCTGA |
| **Mouse primers** | | |
| IL1-A | AGGAGAGCCGGGTGACAGT | ACTCAGCCGTCTCTTCTTCAGAA |
| P53 | TGTTCCGGGAGCTGAATGAG | TGAGCCCTGCTGTCTCCAG |
| P21 | GTGATGTCCGACCTGTTCCG | CGCAACTGCTCACTGTCCAC |
| Cox-2 | TGGTGCCTGGTCTGATGATG | CAAACTTGAGTATGTCGCACACTCT |
| IL-6 | GGTGACAACCACGGCCTTC | AGGTCTGTTGGGAGTGGTATCCT |
| HPRT1 | GGGATTTGAATCACGTTTGTG | TTGCGCTCATCTTAGGCTTT |
| IL-10 | GGAGCATTTGAATTCCCTGG | GAAATCGATGACAGCGCCTC |
| **Rat Primers** | | |
| IL6st | TGATGGGCTATACCCCCATC | GAGACATTCCCAAGGGCATTC |
| COX-2 | ACCCGGACTGGATTCTACGG | TGGGCTTCAGCGGTAATTTG |
| IL-1 | CTGAAGATGACCTGGAGGCC | TGGAAGCTGTGAGGTGCTGAT |
| VEGF-ɑ | AGCGGAGAAAGCATTTGTTTG | TTGCAACGCGAGTCTGTGTT |
| IL-6 | TGTTGTTGACAGCCACTGCC | CTGTTGTGGGTGGTATCCTCTG |
