# Supplementary material for: Downregulation of the inflammatory network in senescent fibroblasts and aging tissues of the long‐lived and cancer‐resistant subterranean wild rodent, Spalax
Source: Aging Cell. 2019 Oct 11;19(1):e13045. doi: 10.1111/acel.13045 (PMC6974727; doi:10.1111/acel.13045)
Supplement: Supplementary file 17 [file ACEL-19-e13045-s017.doc]

**TableS3: Animals and tissue samples used in the in *vivo* study**.

| **issue** | **species** | **age** |  | **unit** | **group** | **species/strain** | **sex** |
| --- | --- | --- | --- | --- | --- | --- | --- |
| Brain | *Spalax* (set1) | 1 |  | years | Young | *galili* 2n=52 | f |
| Liver/Intestine/Brain |  | 3 |  | years | Young | *galili* 2n=52 | f |
| Liver/Intestine |  | 5 |  | years | young | *galili* 2n=52 | m |
| Liver/Intestine/Brain |  | 12 |  | years | old | *galili* 2n=52 | f |
| Liver/Intestine/Brain |  | 17 |  | years | old | *golani* 2n=54 | f |
| Brain | Rat (set2) | 5 |  | months | Young | *galili* 2n=52 | m |
| Liver/Intestine/Brain |  | 5 |  | months | Young | SD | m |
| Liver/Intestine |  | 7 |  | months | Young | SD | m |
| Liver/Intestine/Brain |  | 16 |  | months | old | SD | m |
| Liver/Intestine/Brain |  | 24 |  | months | old | SD | m |
|  |  |  |  |  |  |  |  |

*Age* column: As *Spalax* cannot be bred in captivity, they are captured in the field, and only 1-year old animals or older can be discriminated, ages of all *Spalax* individuals were determined by the time of their capture until sacrificed.
