# Supplementary material for: Downregulation of the inflammatory network in senescent fibroblasts and aging tissues of the long‐lived and cancer‐resistant subterranean wild rodent, Spalax
Source: Aging Cell. 2019 Oct 11;19(1):e13045. doi: 10.1111/acel.13045 (PMC6974727; doi:10.1111/acel.13045)
Supplement: Supplementary file 18 [file ACEL-19-e13045-s018.docx]

**Appendi**x **S1**

**Experimental procedure**

**Staining for SA- β-Gal**

RS and EIS fibroblasts were re-plated before staining (2, 5 × 10 ^4^ cells/well in six-well plates. After 48 h SA-β-Gal activity was determined. The X-Gal stock solution was prepared by dissolving 40mg/ml X-Gal (Invitrogen, Carlsbad, CA) in dimetylformamide immediately before staining. SA-β-Gal staining solution was prepared as follows: 1 mg/ml of X-Gal stock solution was dissolved in phosphate buffered saline containing 5mM potassium ferrocyanide, 5mM potassium ferricyanide, 2mM MgCl2, with adjusted pH 6.0. Cells were fixed using 0.2% glutaraldehyde in PBS for 15 minutes at RT, washed in PBS and incubated in fresh SA-β-Gal staining solution for overnight at 37°C. The cells were checked for development of the blue color under a light microscope. Quantitative analysis was performed using *Image J* software in four independent fields (in triplicates)

**Immunoblotting**

Following the treatments, cells were washed with ice-cold PBS, lysed in RIPA/SDS buffer to which sodium orthovanadate phosphatase inhibitor and Complete Protease Inhibitor were added. Samples were centrifuged, and supernatants were collected. Protein concentrations were determined with the Bradford Assay (Bio-Rad); proteins were then blotted onto nitrocellulose membrane and incubated with primary antibodies diluted 1:1000. Proteins were visualized by a chemiluminescence detection kit for HRP (EZ ECL, Biological Industries) using MyECL Imager (Thermo Scientific) Quantity One software (Bio-Rad).

**Immunofluorescence**

Fibroblasts from early and late passages (replicative senescence) and after etoposide exposure (premature senescence) were seeded in 6-well plate on glass coverslips at ~40k cells/well. Following recovery period of 24-48 hours, cells were processed for γ-H2AX foci as described earlier (Domankevich, Eddini, Odeh, & Shams, 2018). Cells' nuclei were visualized under fluorescent microscope (Leica DMi8, equipped with Leica DFC365FX camera). The images were used for quantification of the foci using FociCounter software. At least 300 nuclei from several random fields were scored. The preparation of cells for staining of NF-kB-p65 and IL-1α was performed in the same manner as described above. Specification of antibodies and dilutions are shown in Table S1, Supplementary information.

**Flow Cytometry**

Cells (5x10^5^ for each probe) were washed twice with PBS, trypsinized, and transferred to 5- ml tubes, then washed 3 times with PBS and centrifuged at 850 x g, Thereafter hypotonic buffer (Sodium citrate 0.1%; Triton 0.1%) was added to pellet of cells followed by Propidium iodide staining (final concentration 25 µg/ml); The PI fluorescence of individual nuclei was recorded by FACSaria (Becton Dickinson, NJ, USA). A total of 10,000 events were acquired and corrected for debris and aggregates.

**Preparation of RNA, cDNA**

Total RNA from freshly washed cells were extracted using RNeasy Mini Kit (QIAGEN) following the manufacturer's instructions. cDNA samples were synthesized using iScript™ (cDNA Synthesis Kit, Bio-Rad).

**Preparation RNA from tissues**

# RNA was extracted from tissues, flash-frozen in liquid nitrogen, and stored in −80 °C. Spalax and rat brain, intestine and liver RNA was extracted by using TRI Reagent (Molecular Research Center, Cincinnati, USA) following the manufacturer’s instructions. All samples were quantified on a Nanodrop®. qPCR, RNA samples were treated with DNase I (DNA-free, Invitrogen), and 1 μg was taken for first-strand cDNA synthesis (iScript, Bio-Rad) in a 20-μl volume. Aliquots of 1 μl of cDNA were used for each real-time PCR reaction.

**Quantitative Real-Time Polymerase Chain Reaction (RT-PCR)**

Species-specific primers were designed for each target by using Primer3 software (Applied BioSystems) based on the published sequences. Relative quantification of gene transcription was performed by using Fast SYBR Green (Applied BioSystems), and 1 µl of cDNA generated from 50 ng total RNA. Serial dilutions of the cDNA with the highest expression level for each target gene were used to build a relative standard curve and to test amplification efficiency for each experiment. Samples were tested in triplicates. The amplification parameters were as follows: 95°C for 20 sec, followed by 40 cycles of 95°C for 3 sec and 60°C for 30 sec. To verify a single product with fixed melting temperature, melting curve protocol was applied. The quantification relied on equal amounts of total RNA used in each sample, and the reliability of this method was tested and confirmed by HPRT1 and actin housekeeping genes for human, mouse and *Spalax* (Fig S12., Supporting Information)

**Enzyme-linked immunosorbent assay (ELISA)** Human IL-6 ELISA kit (R&D systems) was used, according to the instructions of manufacturer. Briefly, cells were treated with 1µg/ml etoposide for 5d, then cells were washed 2*PBSx1 and incubated in DMEM serum free media for 24h.Complete supernatant was collected, (centrifuged at 120 x g for 5 min at RT)

Domankevich, V., Eddini, H., Odeh, A., & Shams, I. (2018). Resistance to DNA damage and enhanced DNA repair capacity in the hypoxia-tolerant blind mole rat Spalax carmeli. *J Exp Biol, 221*(Pt 8). doi:10.1242/jeb.174540
