# Supplementary material for: Downregulation of the inflammatory network in senescent fibroblasts and aging tissues of the long‐lived and cancer‐resistant subterranean wild rodent, Spalax
Source: Aging Cell. 2019 Oct 11;19(1):e13045. doi: 10.1111/acel.13045 (PMC6974727; doi:10.1111/acel.13045)
Supplement: Supplementary file 19 [file ACEL-19-e13045-s019.docx]

**Appendix S2.**

**Legends for figures presented as Supporting Information**

**Fig. S1** **Effects of serial passaging on SA-β-Gal expression and proliferative rate in *Spalax*, mouse and human fibroblasts. (A)** Representative microscope images demonstrating SA**-**β-Gal positive cells in replicative senescent cells (RS). **(B)** Percentage of SA**-**β-Gal positive cells calculated from a total of 300 cells in 4 independent fields for each biological repeat (three independent individuals for *Spalax* and mice cells; three independent experiments for human cells, in triplicates. The mean value of each species is compared to its control cells (Young). ****p*<0.001. **(C)** 25×10³ cells were plated in 12-well plates and counted after 24, 48, 72- and 96-hours Data is presented as mean of two biological repeats (n=2) in triplicates for each time point. Error bars represent SD.

**Fig. S2. The levels of IL1α, IL6 mRNA expression and IL6 secretion in young, RS and EIS fibroblasts of human.** **(A)** Biological replicates of Fig. 6. The levels of mRNA expression were quantified by using qRT-PCR. **(B)** IL6 concentration in CMs (supernatants) of human young and RS fibroblasts. Fibroblasts were cultured in DMEM serum free media 24h before IL6 quantification. Complete supernatant was collected, (centrifuged at 120 x g for 5 min at RT) and processed for analysis by using Human IL-6 ELISA kit (R&D systems). Data from 2 independent experiments are presented.

**Fig S3.** **The levels of IL1a, IL6 and Cox-2 mRNA expression in young, RS and EIS mouse fibroblasts.** Biological replicates of Fig. 5. The levels of mRNA expression were quantified by using qRT-PCR.

**Fig. S4.** **Non-canonical SASP in RS and EIS *Spalax* fibroblasts and effect of MDA-MB-231 CM: the levels of mRNA (biological replicates of data presented in Fig. 5)**. The levels of mRNA expression were quantified by using qRT-PCR. Treatment by MDA-MB-231 CM is described in Experimental Procedures.

**Fig. S5.** **Treatment of RS *Spalax* fibroblasts by MDA-MB-231 CM increased the number of** γH2AX  **foci in the nuclei (A)** The representative microphotographs showing the levels of DSBs in untreated and treated with MDA-MB-231 *Spalax* RS cells **(B)** A graph showing the foci number represented as a Median ± SD of three independent experiments, *** p ≤ 0.001, differences between the levels of foci in *Spalax* control and MDA-MB-231 CM- treated groups. A total of 250 nuclei were counted.

**Fig. S6. Biological replicates of western blot experiments** presented in Fig. 6. (Data of *Spalax* and human). The levels of p-p65, p-p38 and IL1α in *Spalax* fibroblasts (upper), and human fibroblasts **(**lower), undergoing replicative or etoposide-induced senescence. [Densitometry](https://www.sciencedirect.com/topics/neuroscience/densitometry) reflects relative protein quantity normalized to actin.

**Fig. S7. Biological replicates of western blot experiments** presented in Fig. 6 (Data of mice). The levels of protein activation / expression in senescent mouse fibroblasts. Densitometry reflects the relative amount of p-p65, p-p38 and IL1α normalized to actin.

**Fig. S8. Factors secreted by MDA-MB-231 CM induce cytoplasmic retention and surface-membrane localization of IL1α in *Spalax* senescent fibroblasts**. **(A)** Representative images showing *Spalax* RS fibroblasts untreated (upper panel) and treated with MDA-MB-231 CM for 24h (low panel). Treated/ untreated cells were stained with IL1α–FITC antibody and membrane tracker (wheat germ agglutinin, Alexa Fluor® 594 conjugate), nuclei were counterstained with DAPI. Plots demonstrating fluorescent signals in different cellular compartments (right panels) Measurement of fluorescent signal was made using LAS X Life Science Microscope Software with normalization according to background (green is IL1α fluorescence, blue is DAPI, white is membrane tracker). **(B)** Quantitative data showing differences in IL1α distribution in *Spalax* cells treated or not with MDA-MB-231 CM. At least 20 cells were analyzed in both untreated and treated samples. Experiments were repeated 2 times (n=2) in triplicates. Scale bars are 25 µm. RS, replicative senescence; Sp, *Spalax*, MB-231, MDA-MB-231 CM.

**Fig. S9. NF-κB-p65 nuclear localization and re-distribution of IL1α in RS *Spalax* fibroblasts under effect of MDA-MB-231 CM and LPS.** **(A)** Treatment of Spalax RS cells with MDA-MB-231 CM increased nuclear NF- κB p65 content (complimentary data to Fig.6C). **(B)** The microphotographs showing *Spalax* RS fibroblasts untreated (left panel) and treated with LPS (right panel). Cells were plated on glass coverslips in 6-well plates, and on the next day cells were either untreated or treated with LPS (1µg/ml) for 1h, thereafter cells were washed and stained with anti-NF-κB p65 (total) and anti-IL1α, nuclei were counterstained with DAPI. Representative images are shown. Experiment was repeated 2 times (n=2)**.** Scale bars are 25 µm. RS, replicative senescence; Sp, *Spalax*, LPS, lipopolysaccharide.

**Fig. S10.** **GATA4 abundance in young and RS *Spalax* and human fibroblasts and effect of MDA-MB-231 CM.** **(A)** The microphotographs showing GATA4 immunofluorescence in human young and senescent fibroblasts. **(B)** *Spalax* young (left panel) and RS fibroblasts untreated (central panel) and treated with MDA-MB-231 CM (right panel) for 24h. Treated/ untreated cells were stained with GATA4, nuclei were counterstained with DAPI. **(C)** Quantitative data showing differences in GATA4 abundance between human young and RS fibroblasts, and between *Spalax* young, RS untreated and treated with MDA-MB-231 CM. At least 15 cells were analyzed in each group. Fluorescent signals were measured using LAS X Life Science Microscope Software with normalization according to background. Experiment was repeated 2 times (n=2)**.**  * p ≤ 0.05, differences between *Spalax* young and RS; *** p ≤ 0.001, differences between human young and RS; *** p ≤ 0.001, differences between *Spalax* RS and RS + MB-231 CM. Scale bars are 25 µm. RS, replicative senescence; Sp, *Spalax*, MB-231, MDA-MB-231 CM.

**Fig. S11.** **Expression of SASP genes in intestine and brain of young and old *Spalax*** (qRT-PCR data is complimentary for Fig. 7B).

**Fig. S12.** **Expression of SASP genes in intestine and brain of young and old rats** (qRT-PCR data is complimentary for Fig. 7B).

**Fig. S13.** **Normalization of mRNA expression values.** The mRNA quantification relied on equal amounts of total RNA used in each sample. The accuracy of normalization to total RNA was confirmed by HPRT1 and actin housekeeping genes for mouse (A), *Spalax* (B) and human (C).
